# Supplementary material for: Centromeres are hotspots of cytosine methylation epimutations in a filamentous fungus
Source: bioRxiv. 2026 Mar 5:2026.03.03.709258. Preprint. [Version 1] doi: 10.64898/2026.03.03.709258 (PMC13001447; doi:10.64898/2026.03.03.709258)
Supplement: Supplement 1 [file media-1.pdf]

1 Supplementary Information

Supplemental information for the article: Centromeres are hotspots of cytosine methylation epimutations in a filamentous fungus. By Mariana Villalba de la Peña, Clayton Hull-Crew, Taylor R. Hutter, Cynthia A. Vino, Peter Sarkies, Maria Colomé-Tatché, Frank Johannes, Andrew D. Klocko, and Ilkka Kronholm.

Contents

1 Supplementary Information **1**

1.1 Supplementary methods 2

1.1.1 Chromatin immunoprecipitation 2

1.1.2 Analysis of H3K9me3 divergence 2

1.1.3 Haploid model for estimating epimutation rates 3

1.1.4 Calling genetic mutations from the Nanopore data 4

1.2 Supplementary results 4

1.2.1 Analysis of divergence outliers in WGBS data 4

1.2.2 DMRs in different classes of transposable elements 5

1.2.3 Initial DNA methylation divergence in the WGBS data for centromeric regions 6

1.2.4 Spontaneous changes in H3K9me3 8

1.3 Supplementary Figures 10

1.4 Supplementary Tables 36

1.1 Supplementary methods

1.1.1 Chromatin immunoprecipitation

Strains were grown in VM media overnight at 32 °C. Mycelial pellets were harvested with a Büchner filter, crosslinked with 1% formaldehyde (Sigma Aldrich) in PBS for 15 minutes at room temperature, washed with PBS, and sonicated in ChIP Lysis Buffer (50 mM HEPES, pH 7.5; 140 mM NaCl; 1 mM EDTA; 1% Triton X-100) and Halt Protease and Phosphatase Inhibitor Cocktail (Thermo Fisher Scientific, # 78441), for concurrent cell lysis and chromatin shearing in a Bioruptor Pico (Diagenode), with a 15 minute cycle of 30 seconds sonicating 30 seconds off. The α-H3K9me3 antibody (ActiveMotif cat# 39161) was added to cleared lysate and incubated overnight at 4 °C. Protein A/G magnetic agarose beads (ThermoFisher, cat# PI78609), washed with ChIP Lysis Buffer, were added to the lysate and incubated for two hours at 4 °C. Beads were subsequently washed twice with ChIP Lysis Buffer, once with ChIP Lysis Buffer + 0.5 M NaCl, once with LiCl wash buffer (10 mM Tris-HCl pH 8.0; 250 mM LiCl; 5% IGEPAL CA-630; 1 mM EDTA), and DNA was eluted with 125 µl of TES buffer (50 mM Tris-HCl pH 8.0, 10 mM EDTA, 1% SDS). ChIP-DNA was treated with 100 µg/ml RNaseA, 500 µg/ml Proteinase K, extracted once with phenol/chloroform/Isoamyl alcohol (25:24:1), once with chloroform, ethanol-precipitated with 160 µg/ml glycogen added for DNA recovery, and the ChIP-DNA pellet was resuspended in 30 µl TE. The final ChIP-DNA sample was quantified with a Qubit 3.0, using the HS method.

1.1.2 Analysis of H3K9me3 divergence

For the analysis of H3K9me3 divergence using read counts, we first removed all bins that were not in the seven chromosomes of assembly NC12. Then we normalized the read counts following Love et al. (2014). First we calculated geometric mean for each bin, g_b across all samples as

$$g_b = \exp\left(\frac{\sum_{i=1}^{i} \ln x_{b,i}}{n}\right)$$

where $x_{b,i}$ is the read count of bth bin for sample i, and n is the number of samples. Then a size factor was calculated for each sample by first dividing the count of each bin and each sample by g_b of the bth bin, and then taking the median from all ratios for each sample: $s_i = \mathrm{med}\,(x_{b,i}/g_b)$. Then normalized count, $x_{n,i}$ is obtained by dividing each count by the s_i of that sample, as $x_{n,i} = x_i/s_i$. Then the normalized counts were used in the divergence analysis.

1.1.3 Haploid model for estimating epimutation rates

The original model of AlphaBeta assumes diploid data (Shahryary et al., 2020). Since *N. crassa* is haploid we modified the AlphaBeta somatic model to account for haploidy. Calculating divergence remains the same as in the diploid model. Divergence gets a value of 1 if the cytosines or regions being compared differ in their methylation status, and 0 otherwise. What changes from the diploid model is the transition matrix, $\mathbf{G}$, which in the case of haploid data simplifies to

$$\mathbf{G} = \begin{array}{c} \\ c^u, t \\ c^m, t \end{array} \begin{array}{c} \begin{array}{cc} c^u, t+1 & c^m, t+1 \end{array} \\ \begin{pmatrix} 1-\alpha & \alpha \\ \beta & 1-\beta \end{pmatrix} \end{array}$$

where c^u and c^m are unmethylated and methylated states respectively, α is the rate of methylation, and β is the rate of methylation loss. The proportions of methylated and unmethylated cytosines when the system is at dynamic equilibrium, $\pi = (\pi_u, \pi_m)$, can be found using Markov chain theory $\pi = \pi\mathbf{G}$. This implies a system of linear equations

$$(1-\alpha)\pi_u + \beta\pi_m = \pi_u$$

$$\alpha\pi_u + (1-\beta)\pi_m = \pi_m$$

$$\pi_u + \pi_m = 1$$

that can be solved by substituting $\pi_m = 1 - \pi_u$ and solving for π_m and π_u:

$$\pi = \left(\frac{\alpha}{\alpha + \beta}, \frac{\beta}{\alpha + \beta} \right).$$

The equilibrium proportions of methylated and unmethylated cytosines depend only on rates of methylation gain and loss.

The haploid model also has one parameter less than the diploid model, as there is no proportion of epiheterozygotes in the model. Estimation of the rate parameters was done as in Shahryary et al. (2020), with numerical estimation using the Nelder-Mead algorithm.

1.1.4 Calling genetic mutations from the Nanopore data

Four our previous work, we know the spontaneous genetic mutations that had happened in the final MA transfer. These mutations were called using Illumina sequencing, and we have validated genotyping pipeline using different methods, including validating a set of the mutations by Sanger sequencing (Villalba de la Peña et al., 2023). To call these mutations from the intermediate MA transfers from the Nanopore-seq dataset we simply examined the read alignment of each sample in IGV. Our observation is that when Nanopore basecalling has been performed using the super accurate model in Dorado, real SNPs can be identified from the alignments unambiguously. Indeed, when we compared the Illumina alignment from Villalba de la Peña et al. (2023) with the Nanopore alignments at positions where the known mutations occur, we could identify the transfers where this mutation appeared (Figure S25).

1.2 Supplementary results

1.2.1 Analysis of divergence outliers in WGBS data

We initially observed that the variance in divergence among the WGBS samples increased substantially after approximately 500 mitoses, particularly for single cytosines (Figure 2). We examined the possibility that this phenomenon was caused by one or few samples that were outliers, due to a

technical issue in the library preparation for example, and all pairwise comparisons to such outlier samples were biased as a result. We first looked at the data for single cytosines in CG context: we chose an arbitrary threshold of divergence of 0.00175 as bulk of the data seemed lie below this threshold, and asked were there particular samples that were associated with pairwise comparisons that exceeded this threshold (Figure S3). We identified four samples out of 69 that were associated with pairwise comparisons with extreme values: Line 13 transfer 20, Line 6 transfer 40, Line 35 transfer 20, and Line 36 transfer 40 (Figure S3). Removing these samples from the data removed nearly all pairwise comparisons with extreme values. However, increase in variance after 500 mitoses could still be observed even after these outlier samples were removed (Figure S3).

We then fitted an AlphaBeta model of epimutation accumulation to the data where we had excluded the four outlier samples described above (Figure S4). Removing these outlier samples does reduce the variance compared to the full dataset, particularly for DMR divergence (Figure 2). However, in all cases the model still does not fully capture the saturation effect in the data (Figure S4). A model of neutral accumulation of epimutations is preferred over null model of no accumulation for single cytosines: $p < 2.2 \times 10^{-16}$ in CG, CHG, and CHH contexts. The results were similar for DMRs: the model of neutral epimutation accumulation was preferred over the null model: $p < 2.2 \times 10^{-16}$ in all sequence contexts.

1.2.2 DMRs in different classes of transposable elements

We investigated whether different types of TEs in centromeric and non-centromeric regions could in principle explain why centromeric regions have so much more DMRs. It could be that TE composition in centromeric and non-centromeric region is different and the TE classes present in centromeric regions are especially prone for DNA methylation changes. To examine this possibility, we first checked which classes of TEs are present in centromeric and non-centromeric regions (Figure S5A). There were some differences in TE composition of centromeric and non-centromeric regions: DNA transposons were more common in non-centromeric regions, even this class is very rare in the *N. crassa* genome. Furthermore, SINEs and unknown elements were also more common

in non-centromeric regions, with TEs of unknown class representing the largest difference of 18.1 percentage points between non-centromeric and centromeric regions (Figure S5A). LINE/Tad1 elements, LTRs, Helitrons, and non-LTE elements were common in centromeric regions. However, the difference between centromeric and non-centromeric regions for the second most common class of LTR elements was only 6.6 percentage points, and 11.3 percentage points for LINE/Tad1 elements (Figure S5A).

The second step was to examine whether DMRs were more common in certain TE classes, we fit a model DMR occurrence rates for the different TE classes across the genome. We did observe that DMRs occurred more often in some TE classes relative to random expectation. DNA transposons had lower rates of DMR occurrance than expected based on their abundance, and the same was true for SINE elements (Figure S5B). DMRs that had higher rates of occurrance in LINE/Tad1, Helitrons, and non-LTR class elements, while DMRs occurred at expected levels in LTR elements (Figure S5B).

To assess whether these differences in TE composition between centromeric and non-centromeric regions and differences in DMR occurrence in different TE classes could explain the differences in amounts of observed DMRs in TEs between centromeric and non-centromeric regions, we used the previous model of DMR rates in different TE classes to predict the amounts of DMRs in centromeric and non-centromeric TEs, given the TE composition of these regions. The model did not predict the observed DMR counts correctly (Figure S5C). This means that much higher amount of DMRs observed in centromeric TEs cannot be explained by TE composition differences alone, and we need to invoke some other centromere specific factor.

1.2.3 Initial DNA methylation divergence in the WGBS data for centromeric regions

While we consider that we get the most precise estimates of epimutation rates from the Nanopore-seq dataset, it is nevertheless interesting to check whether we obtain comparable estimates from the WGBS dataset. Since the saturation effect was not captured well by the epimutation model, we examined only the initial 300 mitoses where divergence in the centromeric regions looks more

linear in the WGBS dataset (Figure S6 and S7).

For single sites divergence increases in a nearly linear fashion for the first 300 mitoses (Figure S10). Neutral epimutation accumulation model was preferred over the null model of no accumulation in all sequence contexts: $p = 3.29 \times 10^{-6}$ for CG, $p = 8.93 \times 10^{-11}$ for CHG, and $p = 1.67 \times 10^{-6}$ for CHH context. The epimutation rate estimates we obtained for single sites were roughly comparable to the estimates we obtained from the Nanopore-seq dataset. For CG context we obtained $\alpha = 1.29 \times 10^{-5}$ and $\beta = 6.16 \times 10^{-5}$, so α is consistent with Nanopore dataset and β is slightly lower. For CHG context we obtained $\alpha = 1.40 \times 10^{-5}$ and $\beta = 7.79 \times 10^{-5}$, which are both consistent with Nanopore dataset estimates. For CHH context we obtained $\alpha = 1.03 \times 10^{-5}$ and $\beta = 5.85 \times 10^{-5}$, so α is lower than our Nanopore dataset estimate while β is consistent. Overall, for single sites the epimutation rate estimates are mostly consistent between the two datasets and differences are within measurement uncertainty (Table 1).

For DMRs we observed a somewhat different picture than for single sites, while divergence for CG context is nearly linear for the first 300 mitoses, for CHG and CHH contexts there seems to be more variance (Figure S10 and S6). Neutral epimutation accumulation model was preferred over the null model of no accumulation in CG context ($p = 1.07 \times 10^{-11}$), in CHG context but not as strongly ($p = 1.17 \times 10^{-3}$), and there was no evidence that accumulation was preferred over the null model in CHH context ($p = 0.19$). The epimutation rate estimates for DMRs were generally not consistent with the Nanopore-seq dataset. For CG context we obtained $\alpha = 6.17 \times 10^{-5}$ which is higher than our Nanopore dataset estimate, and $\beta = 25.60 \times 10^{-5}$ which is also higher. For CHG context we obtained $\alpha = 3.98 \times 10^{-5}$ which is higher than our Nanopore dataset estimate, and $\beta = 1.70 \times 10^{-3}$ which is considerably higher than our Nanopore dataset estimate. For CHH we observed that $\alpha = 4.61 \times 10^{-5}$ which is not too far off from our Nanopore dataset estimate, but $\beta = 1.16 \times 10^{-3}$ which is considerably higher than our Nanopore dataset estimate (Table 1). For CHG and CHH contexts we also observed that the model had numerical convergence issues. There seems to be something in the CHG and CHH contexts in the WGBS DMR dataset that causes strange divergence values for these time points, which in turn leads to problems in epimutation rate

estimation.

1.2.4 Spontaneous changes in H3K9me3

When we first analyzed divergence in H3K9me3 read counts we observed that divergence had a very slight negative slope (Figure S21A). If changes in H3K9me3 patterns are transmitted across mitoses the MA lines should diverge from each other and slope of divergence should be positive, and a negative slope does not have a clear biological interpretation. We investigated further what could cause this, and when we plotted divergence that each sample had to other samples within a pedigree we observed that there were two samples that had high divergence to all other samples (Figure S21B). Both of these samples were replicates of the mat A ancestor. Two other replicates of this ancestor did not exhibit the same pattern. This suggested that there could be a technical reason instead, perhaps some bias that occurred during library preparation or sequencing. When we excluded these two samples, slope of the divergence was not different from zero (Figure S21C). We also observed that and when we performed the analysis separately for the two different MA pedigrees, with all samples included, we did not observe significant divergence. For the mat *A* pedigree: $\beta = -0.096$, $p = 0.058$ and for the mat *a* pedigree: $\beta = -0.004$, $p = 0.817$. Since we observed that DNA methylation changes were transmitted in the centromeric regions, we analyzed the data also by using only 500 bp bins in the centromeric regions, but we did not observed significant different (Figure S21D) nor was there divergence in regions outside the centromeres (Figure S21E).

Supplementary References

Love, M. I., Huber, W., and Anders, S., 2014. Moderated estimation of fold change and dispersion for RNA-seq data with DESeq2. *Genome Biology*, **15**:550.

Shahryary, Y., Symeonidi, A., Hazarika, R. R., Denkena, J., Mubeen, T., Hofmeister, B., van Gurp, T., Colomé-Tatché, M., Verhoeven, K. J. F., Tuskan, G., *et al.*, 2020. AlphaBeta: computational

inference of epimutation rates and spectra from high-throughput DNA methylation data in plants. *Genome Biology*, **21**(1):260.

Villalba de la Peña, M., Summanen, P. A. M., Liukkonen, M., and Kronholm, I., 2023. Chromatin structure influences rate and spectrum of spontaneous mutations in *Neurospora crassa*. *Genome Research*, **33**:1–13.

1.3 Supplementary Figures

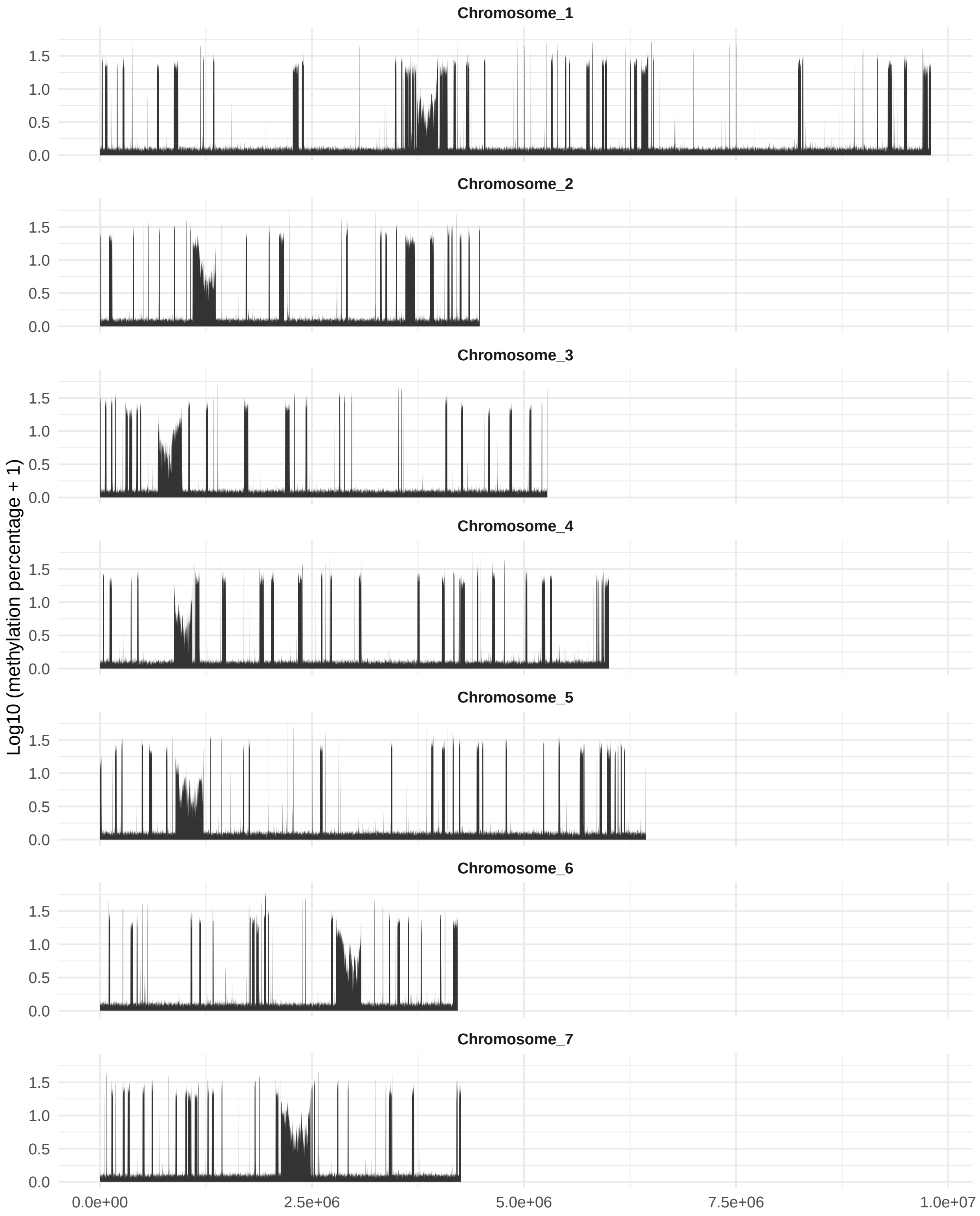

Figure S1: DNA methylation levels across the seven chromosomes of *N. crassa*. Each chromosome was divided into 1 kb bins, which are represented as genomic positions on the x-axis. The y-axis shows the percentage of DNA methylation (note the $\log_{10}$ scale).

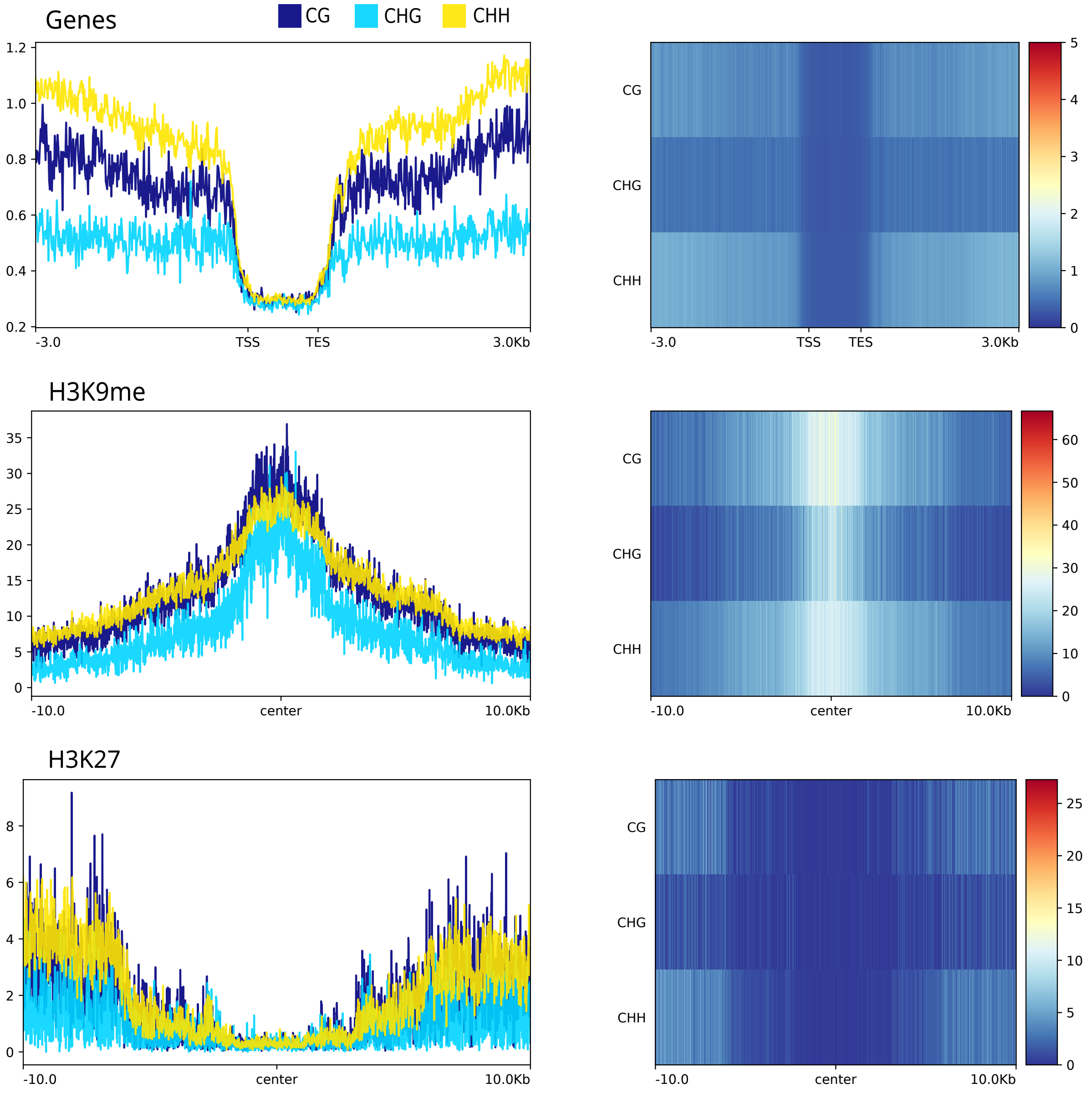

Figure S2: Metaplots and heatmaps showing the percentage of DNA methylation (y-axis) around transcription start sites (TSSs) and transcription end site (TES) (top), within 10 kb of the centers of H3K9me3 regions (middle), and within 10 kb of the centers of H3K27me3 regions (bottom).

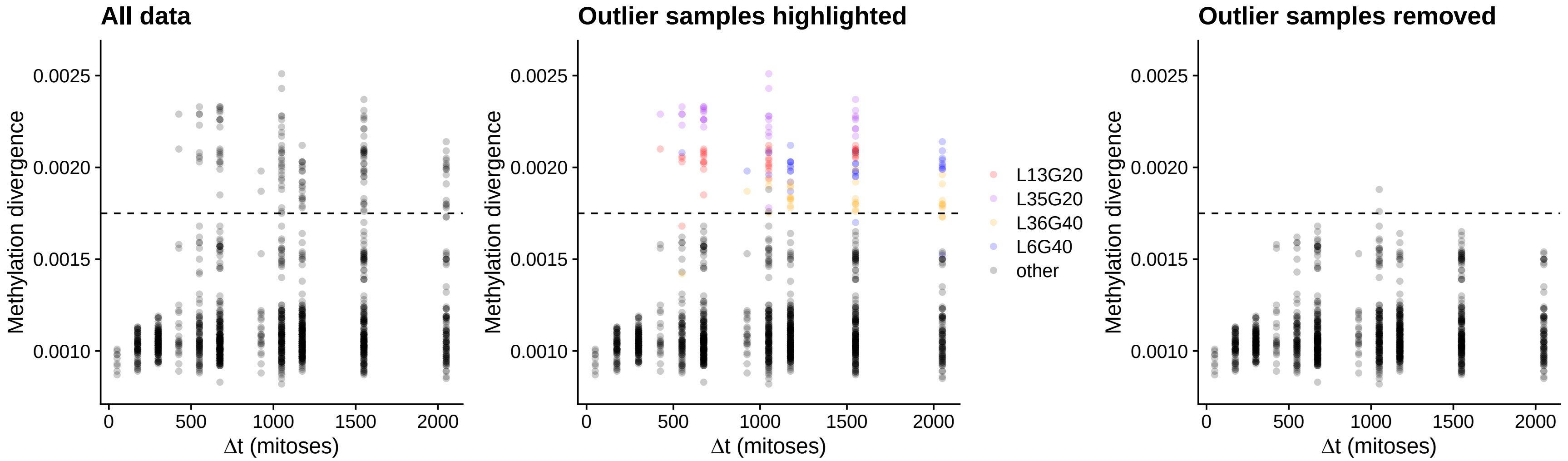

Figure S3: Divergence among the MA lines at single cytosines in CG context for the WGBS dataset. Divergence values above threshold 0.00175, showed by dashed line, were considered outliers. Then samples associated with the outlier pairwise comparisons were identified, and four samples: Line 13 transfer 20, Line 6 transfer 40, Line 35 transfer 20, and Line 36 transfer 40 were observed to be associated with multiple outlier comparisons. Removing these samples from the data removes nearly all extreme divergence values.

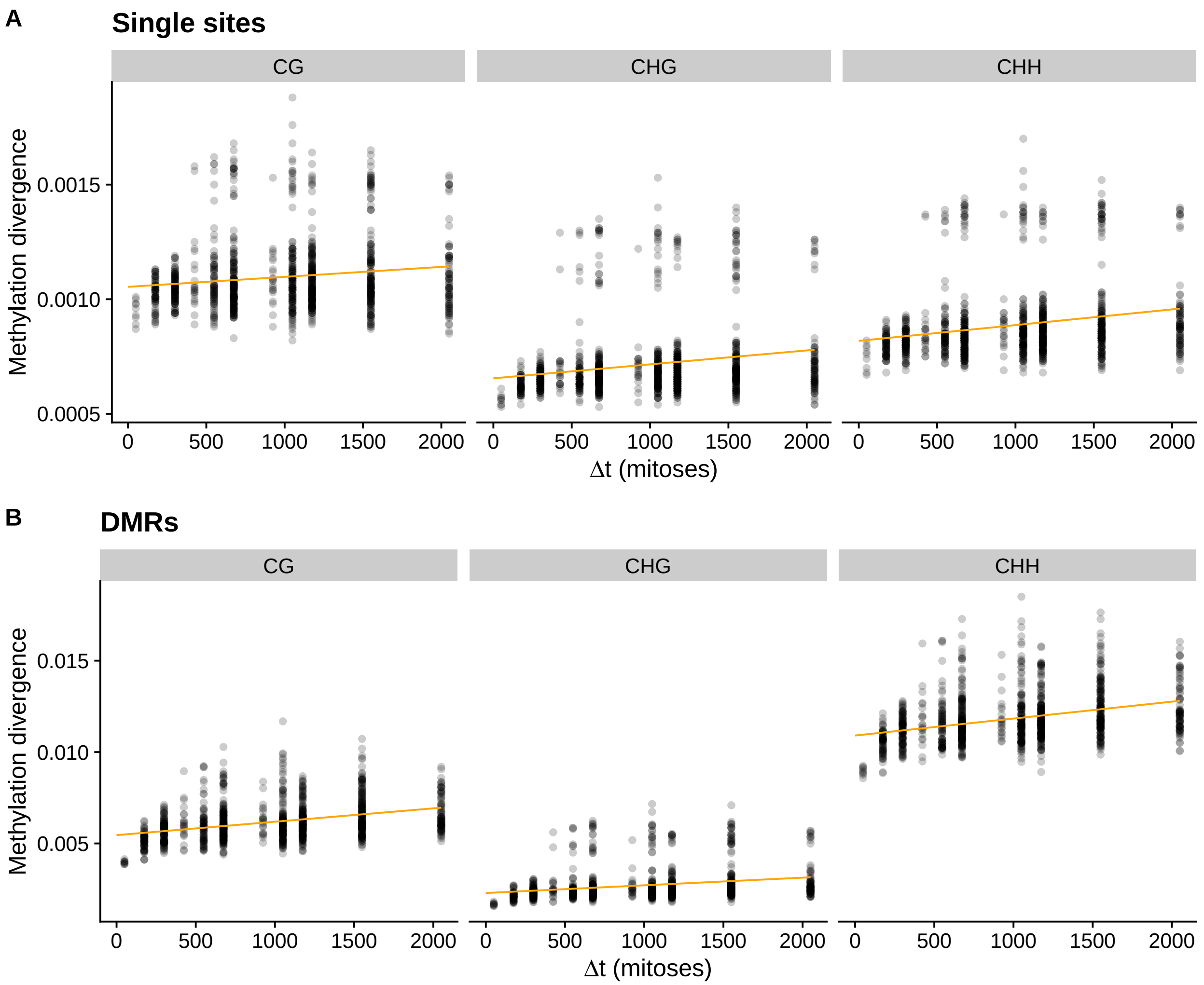

Figure S4: Divergence in cytosine methylation patterns among the MA lines and their ancestors. Outlier samples (Figure S3) have been removed from the data. X-axis shows the number of mitoses separating the two lines that are being compared, and the y-axis shows cytosine methylation divergence. Orange line shows a neutral AlphaBeta model fit. A) The divergence calculated from single cytosines, for CG, CHG, and CHH sequence motifs. B) The divergence calculated for DMRs in different sequence contexts, as in panel A.

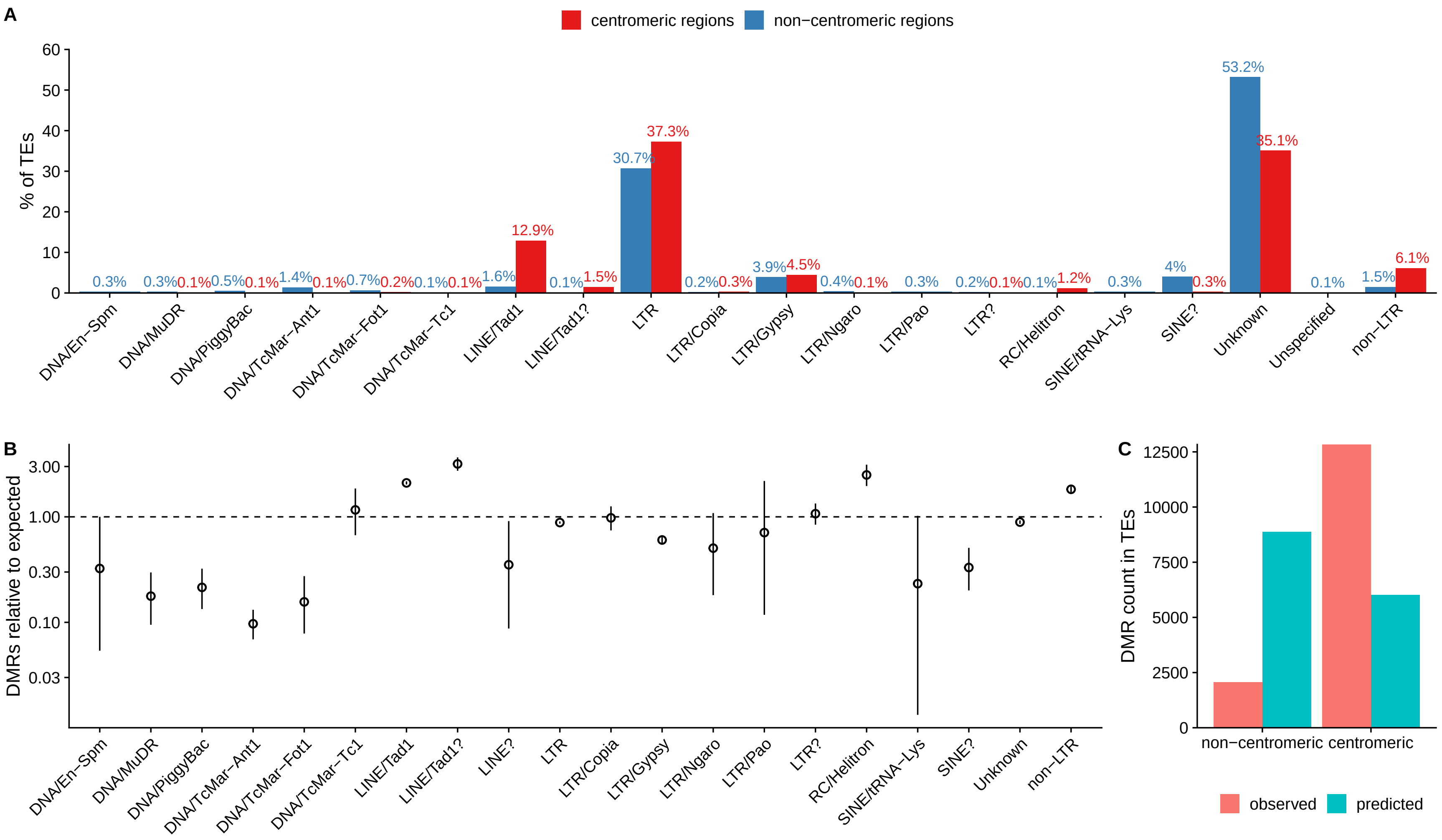

Figure S5: Different TE classes and DMRs. A) Distribution different TE classes among in centromeric and non-centromeric regions. Classes that represented $< 0.1\%$ of TE's were not plotted. B) Rates of DMR occurrence in different TE classes across the whole genome, relative to random expectation. Two exceptionally rare classes of TEs with no DMRs were not plotted as their estimates had large uncertainty. C) Observed and predicted amounts of DMRs, given the distribution of TEs observed in centromeric and and non-centromeric regions (A) and rates od DMRs in these TE classes (B).

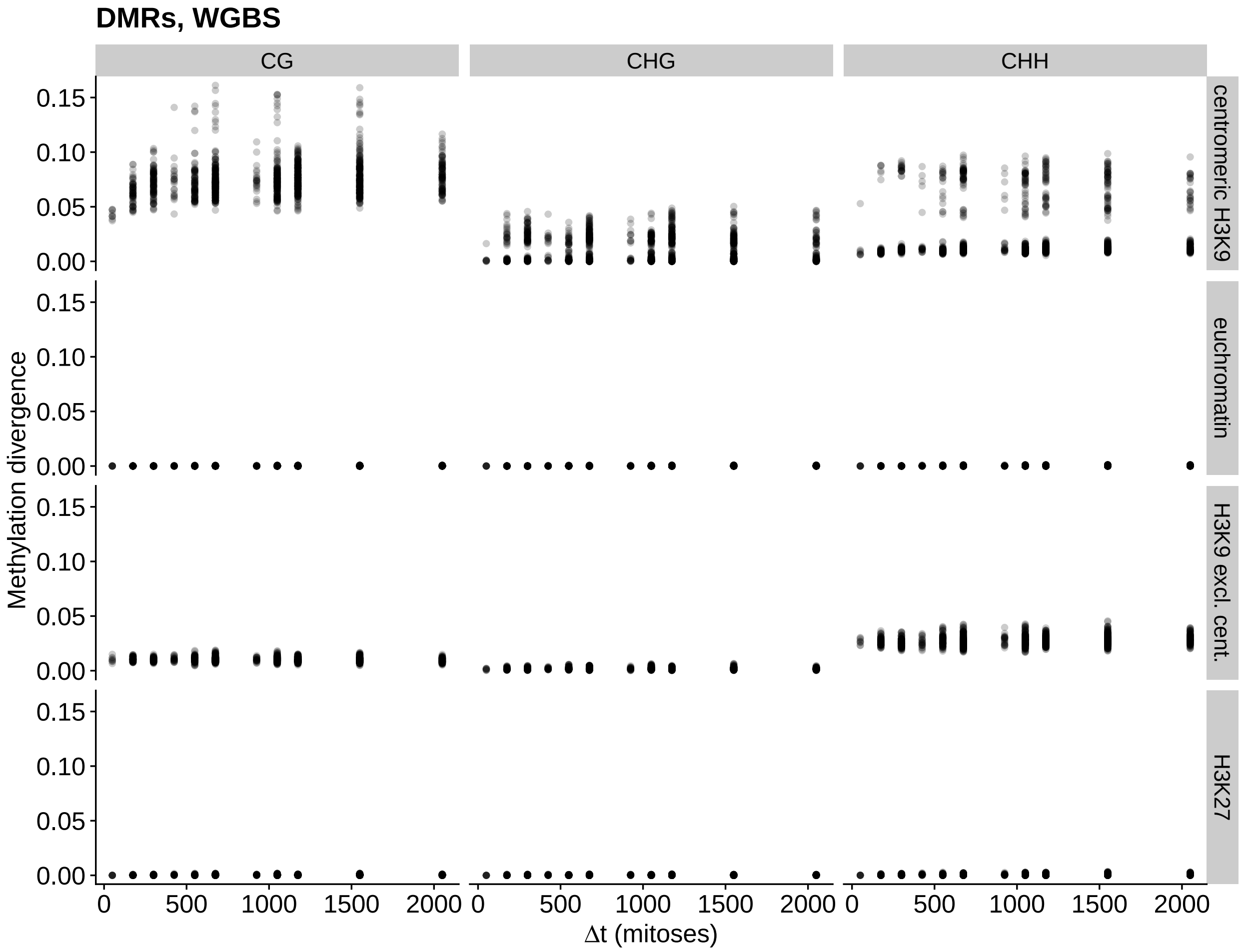

Figure S6: Pairwise divergence in methylation patterns among the MA lines and their ancestors for different regions of the genome. Methylation was detected with WGBS and divergence was calculated for DMRs. X-axis shows the number of mitoses separating the two lines that are being compared, and the y-axis shows methylation divergence.

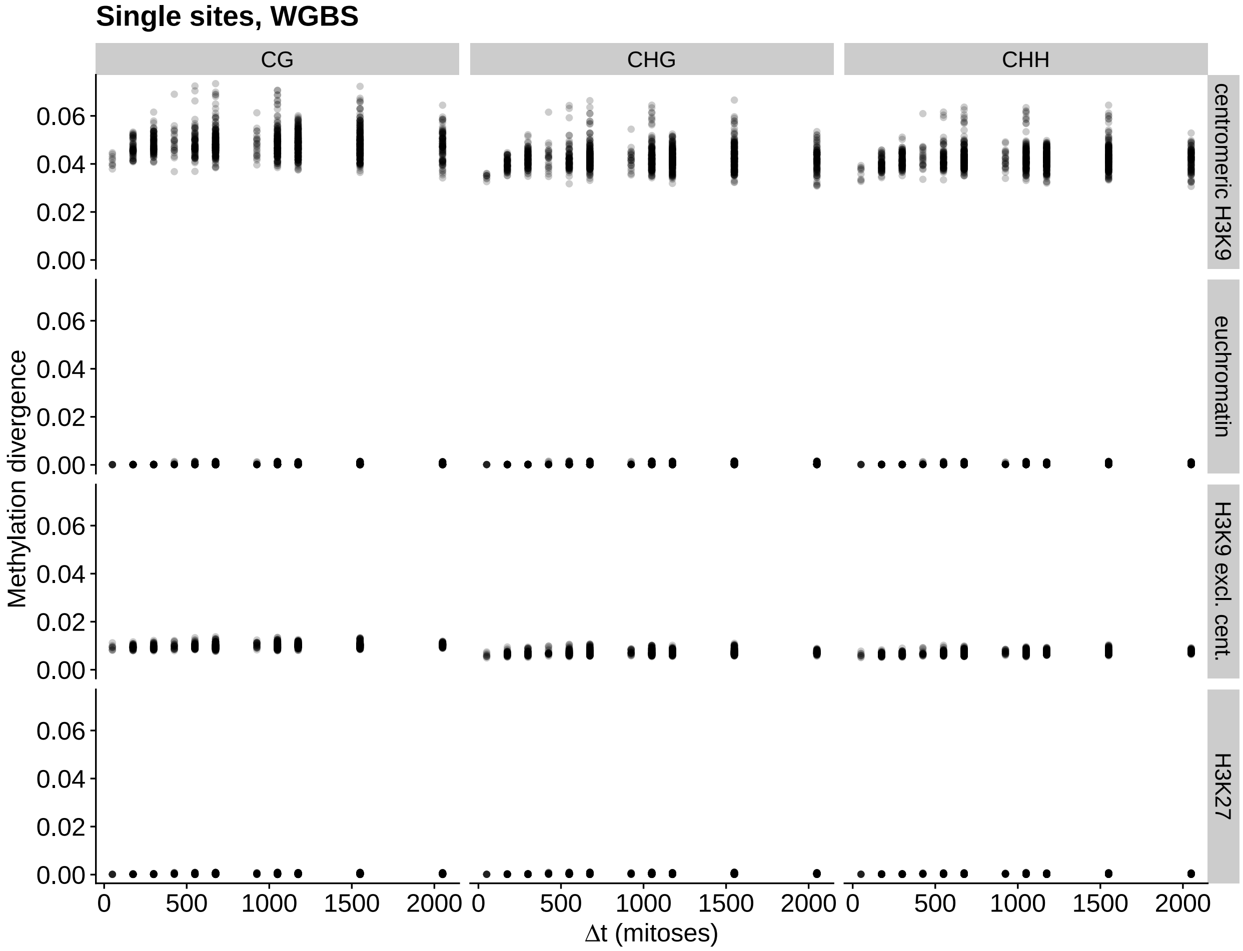

Figure S7: Pairwise divergence in methylation patterns among the MA lines and their ancestors for different regions of the genome. Methylation was detected with WGBS and divergence was calculated for single cytosines. X-axis shows the number of mitoses separating the two lines that are being compared, and the y-axis shows methylation divergence.

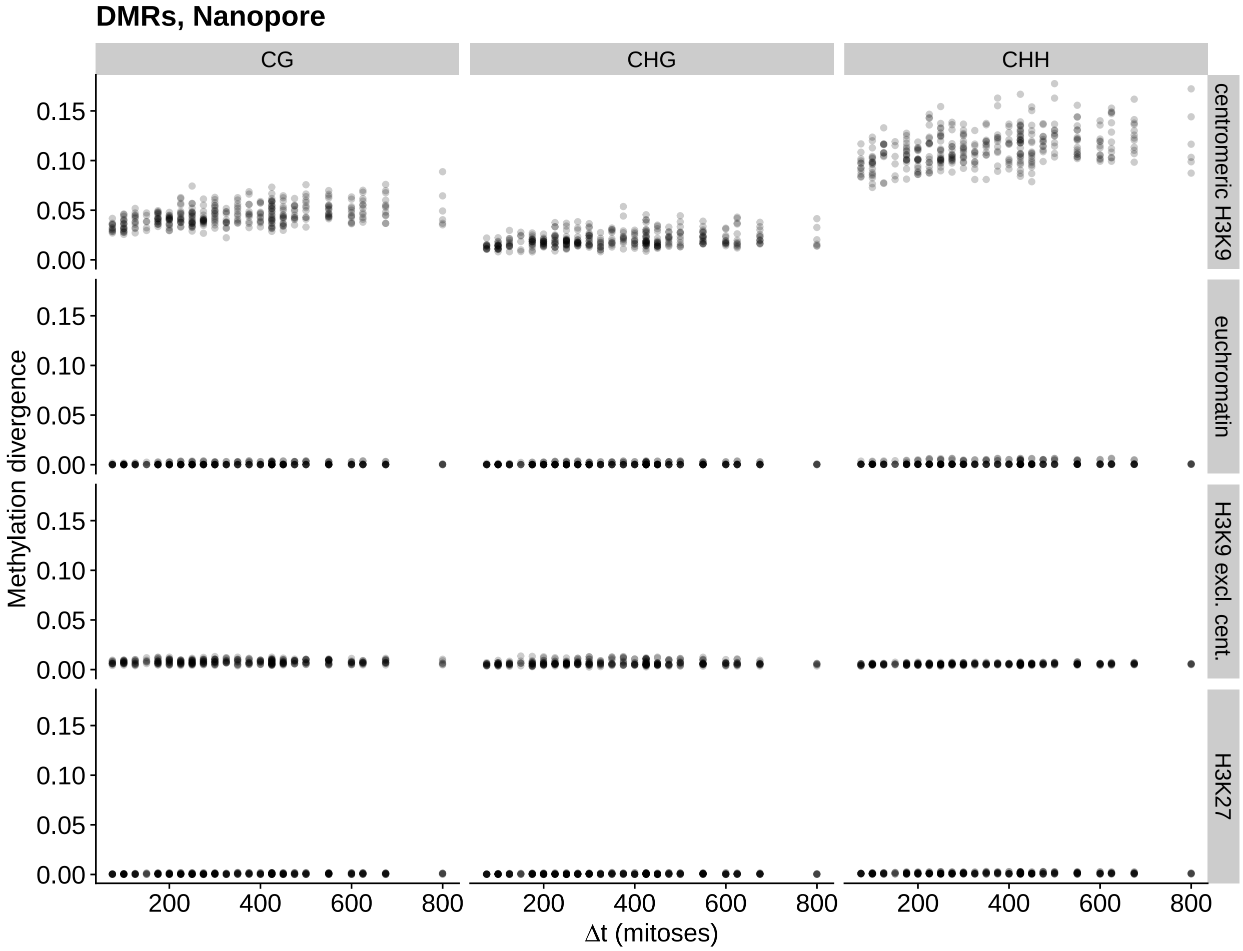

Figure S8: Pairwise divergence in methylation patterns among the MA lines and their ancestors for different regions of the genome. Methylation was detected with Nanopore sequencing and divergence was calculated for DMRs. X-axis shows the number of mitoses separating the two lines that are being compared, and the y-axis shows methylation divergence.

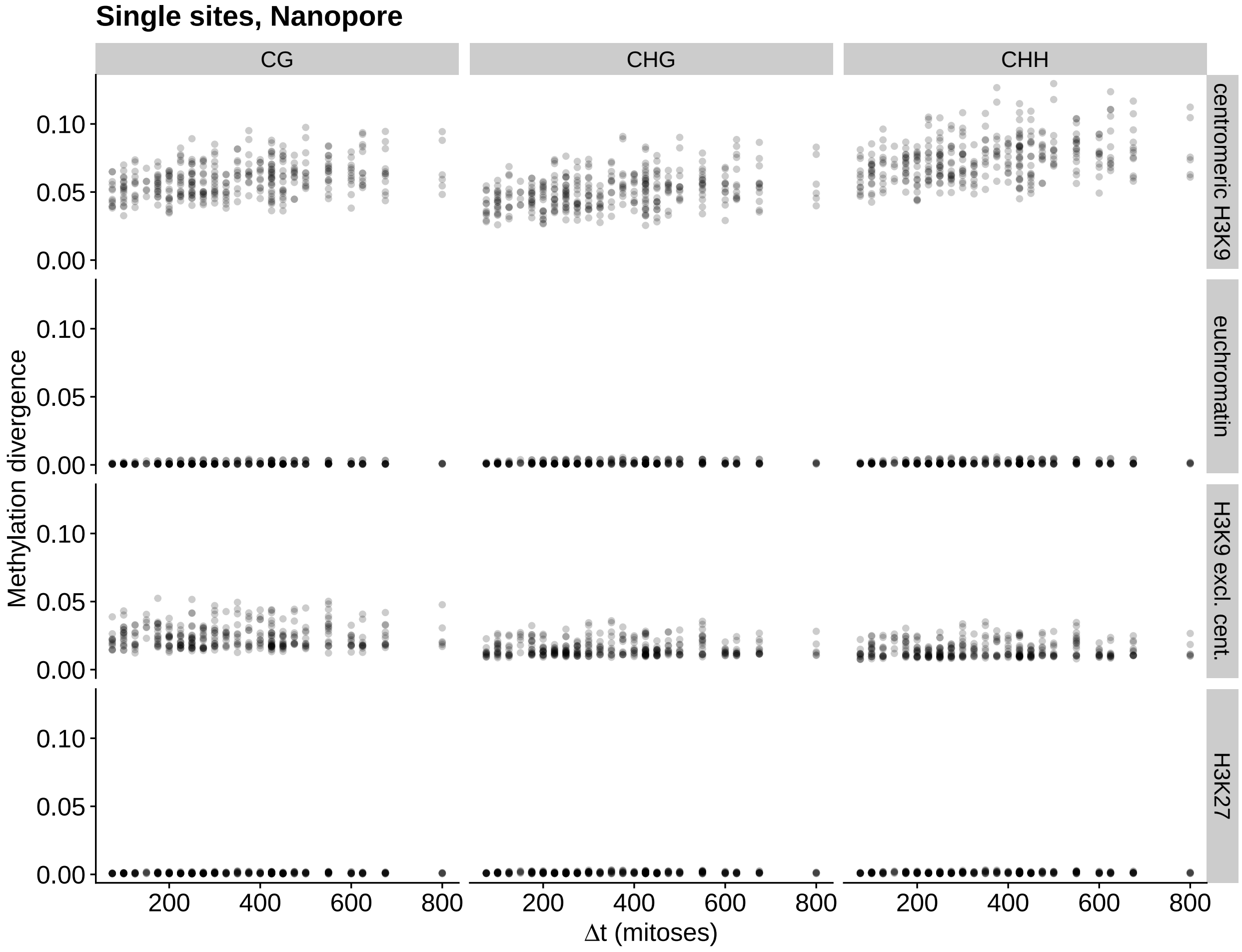

Figure S9: Pairwise divergence in methylation patterns among the MA lines and their ancestors for different regions of the genome. Methylation was detected with Nanopore sequencing and divergence was calculated for single cytosines. X-axis shows the number of mitoses separating the two lines that are being compared, and the y-axis shows methylation divergence.

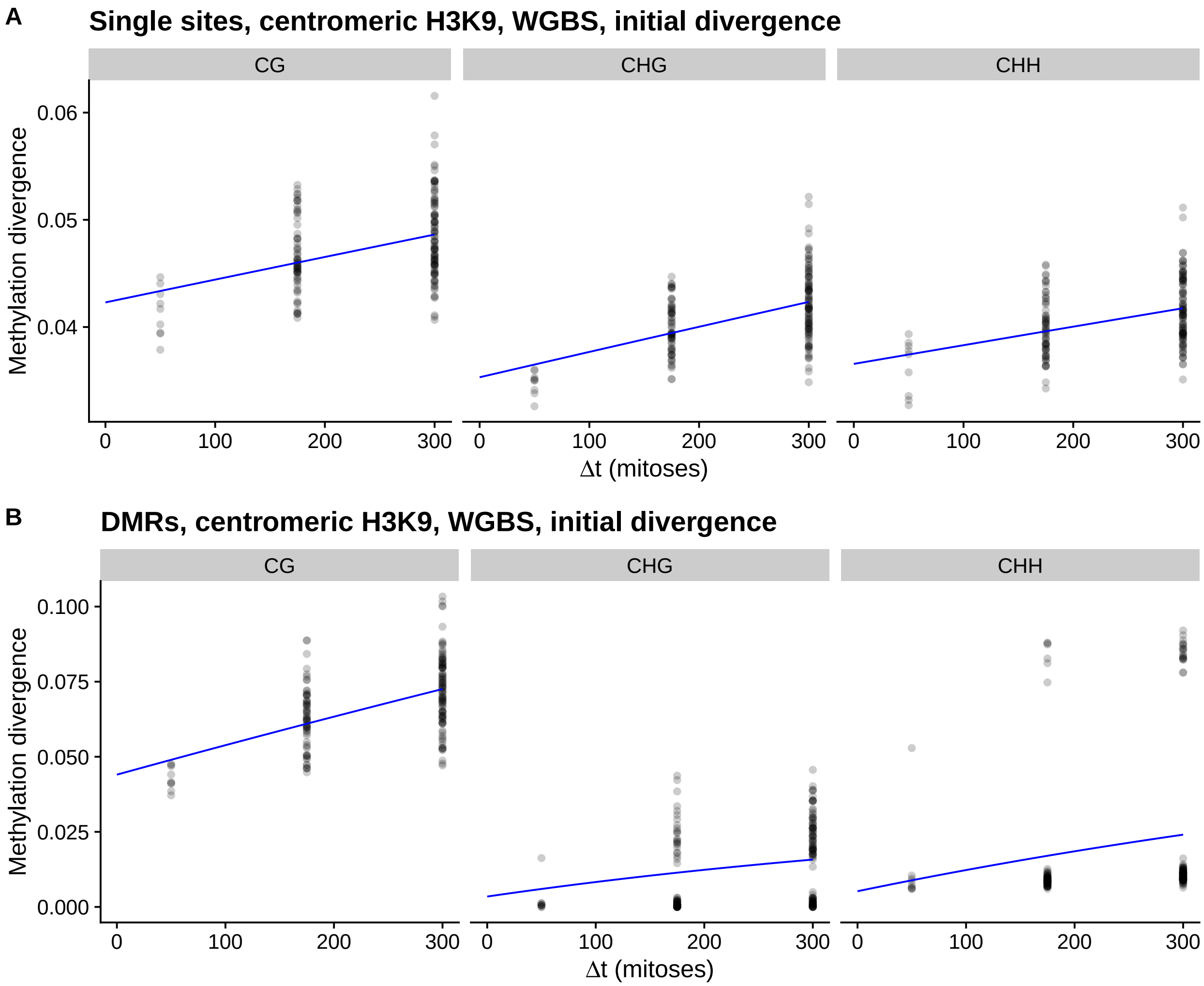

Figure S10: Pairwise divergence in methylation patterns in centromeric regions among the MA lines and their ancestors in the WGBS dataset only for the initial 300 mitoses. Blue line is a fit from the AlphaBeta neutral epimutation accumulation model. A) Divergence calculated for single sites. B) Divergence calculated for DMRs.

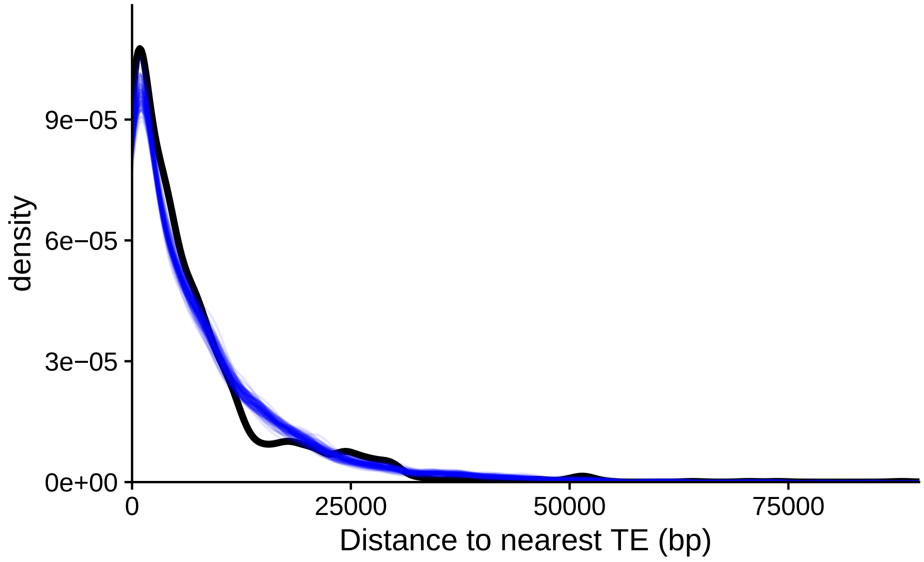

Figure S11: Black line shows the density of distances from DMRs that occurred in euchromatic genes and promoters to the nearest TE. There were 1694 DMRs in euchromatic genes, and 549 in euchromatic promoters. Blue lines represent the density of distances from random euchromatic genes and promoters to the nearest TE. One hundred independent simulations were performed, with the same number of genes and promoters sampled from euchromatin.

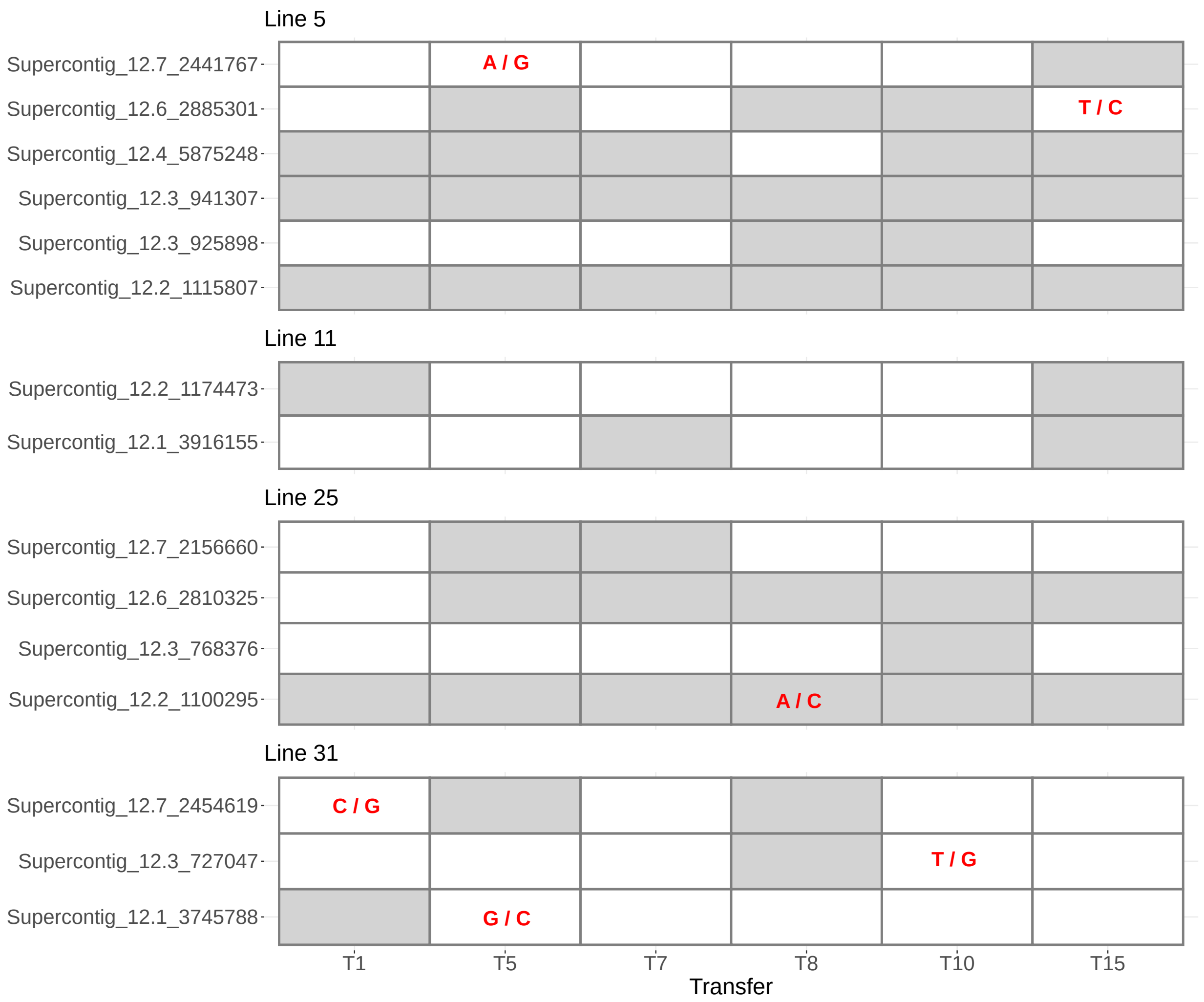

Figure S12: Differentially methylated regions (DMRs) that overlap with genetic mutations are depicted in rows and columns indicate different transfers of the MA experiment. Grey and white colours indicate different methylation states for that DMR. A genetic mutation occurring in a particular transfer is marked in red (ancestor base/derived base). Rows without genetic mutations signify DMR locations where the genetic mutation occurred after transfer fifteen, outside our Nanopore-seq dataset.

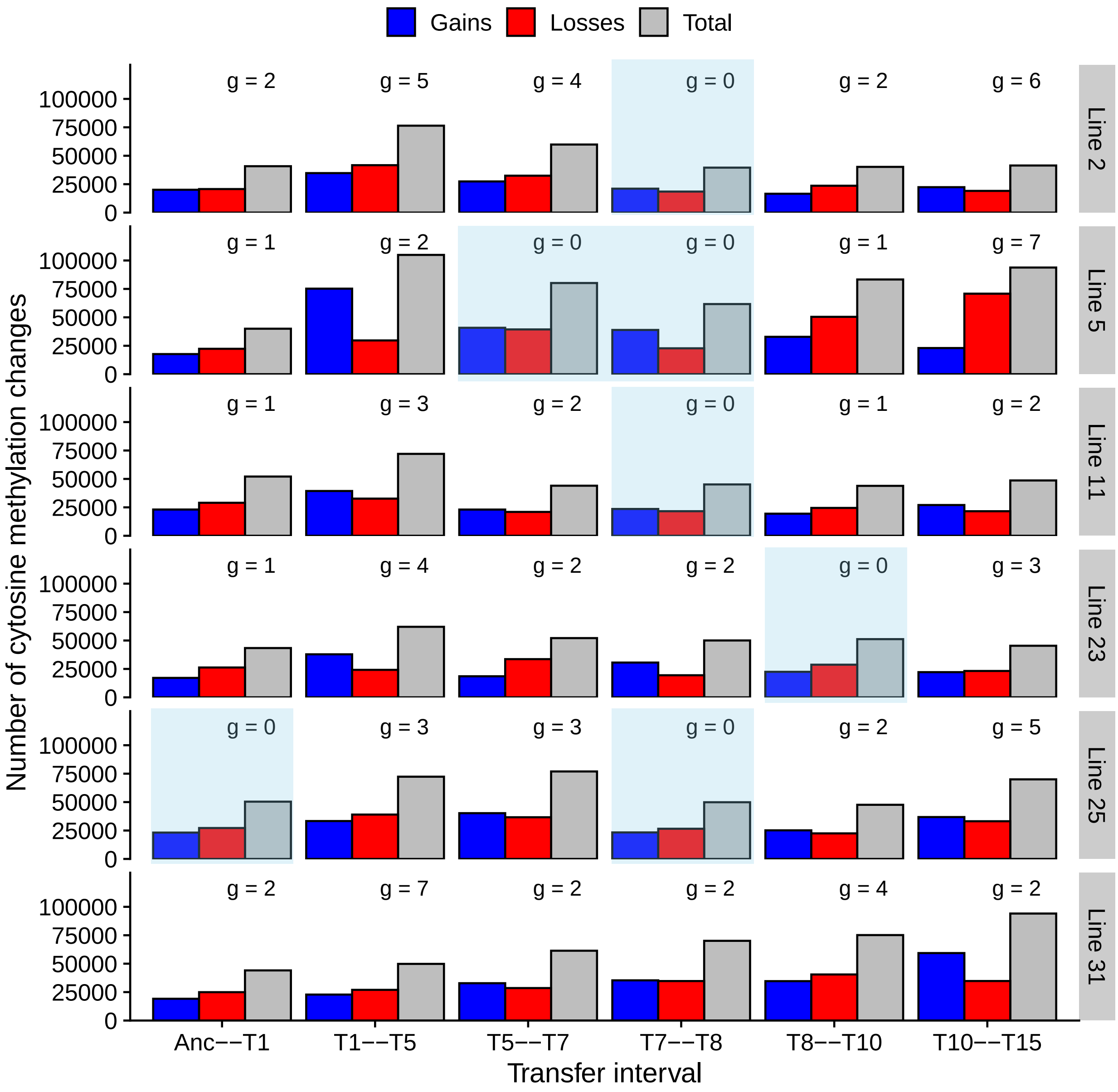

Figure S13: Number of cytosine methylation changes at each interval for the samples sequenced with Nanopore. Number of genetic mutations that happened in each interval is shown on top of the bars and intervals where no genetic mutations occurred are highlighted in blue.

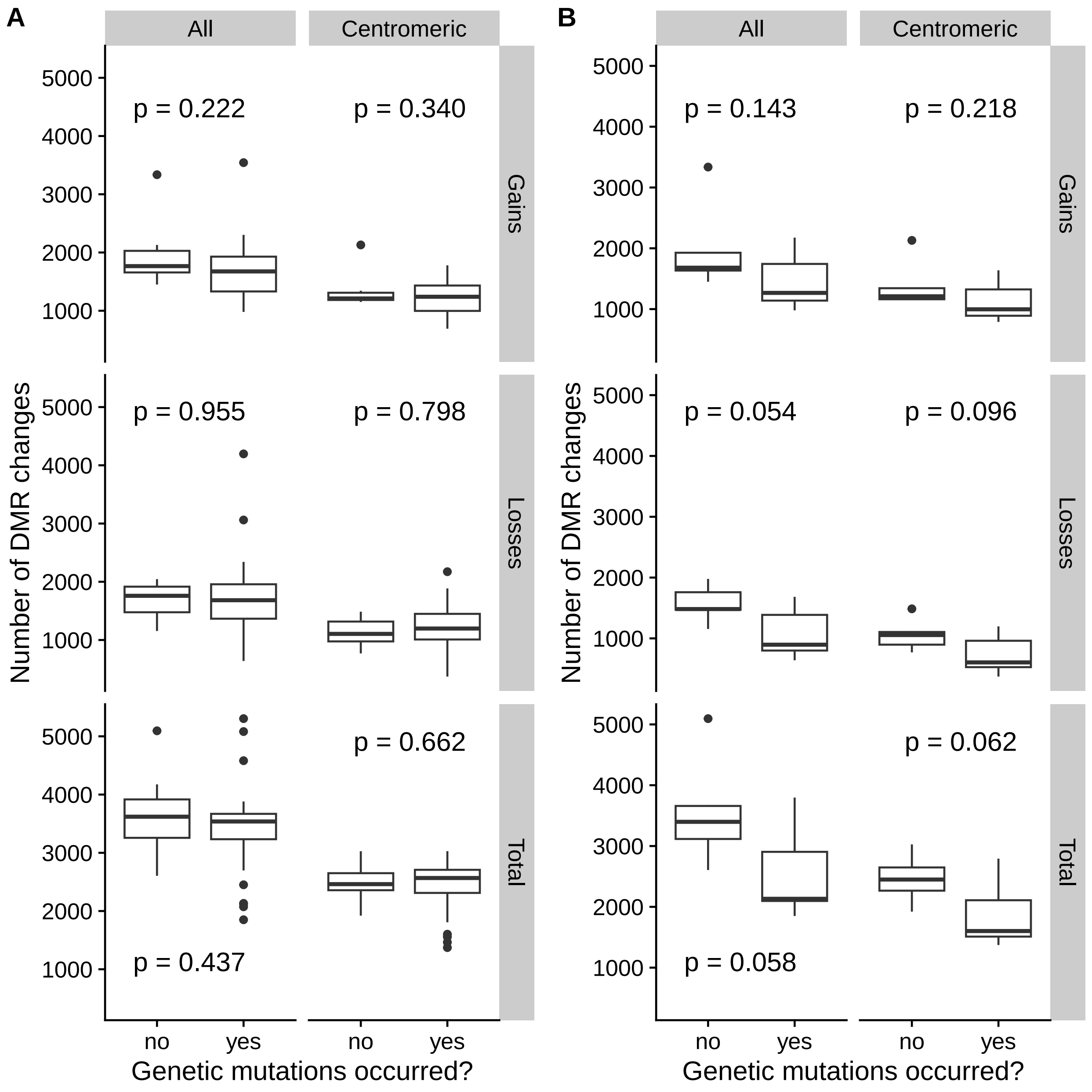

Figure S14: A) Number of DMR changes for each interval and whether genetic mutations occurred in that interval for the Nanopore-seq dataset. There were 36 intervals, and 7 intervals without genetic mutations. B) Only those intervals that contained a single transfer. There were 12 such intervals, of which five did not have a genetic mutation.

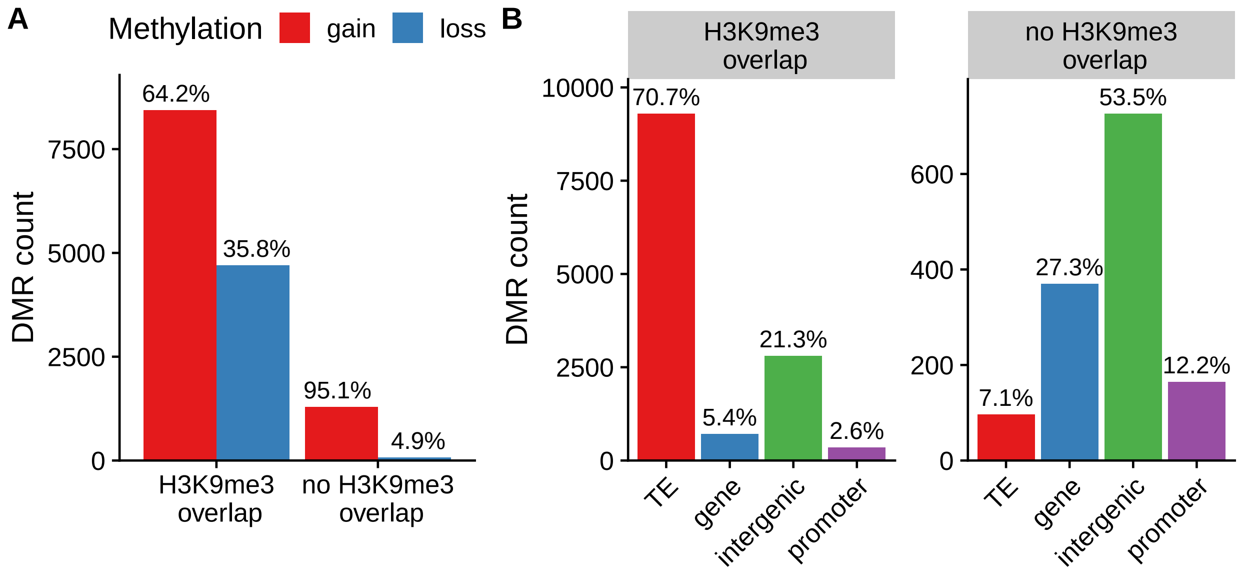

Figure S15: Properties of DMRs and whether they overlap H3K9me3. A) DMR counts and whether the DMR resulted in gain or loss of methylation for non-overlapping and for those overlapping with H3K9me3. B) Annotations of DMRs overlapping or non-overlapping H3K9me3.

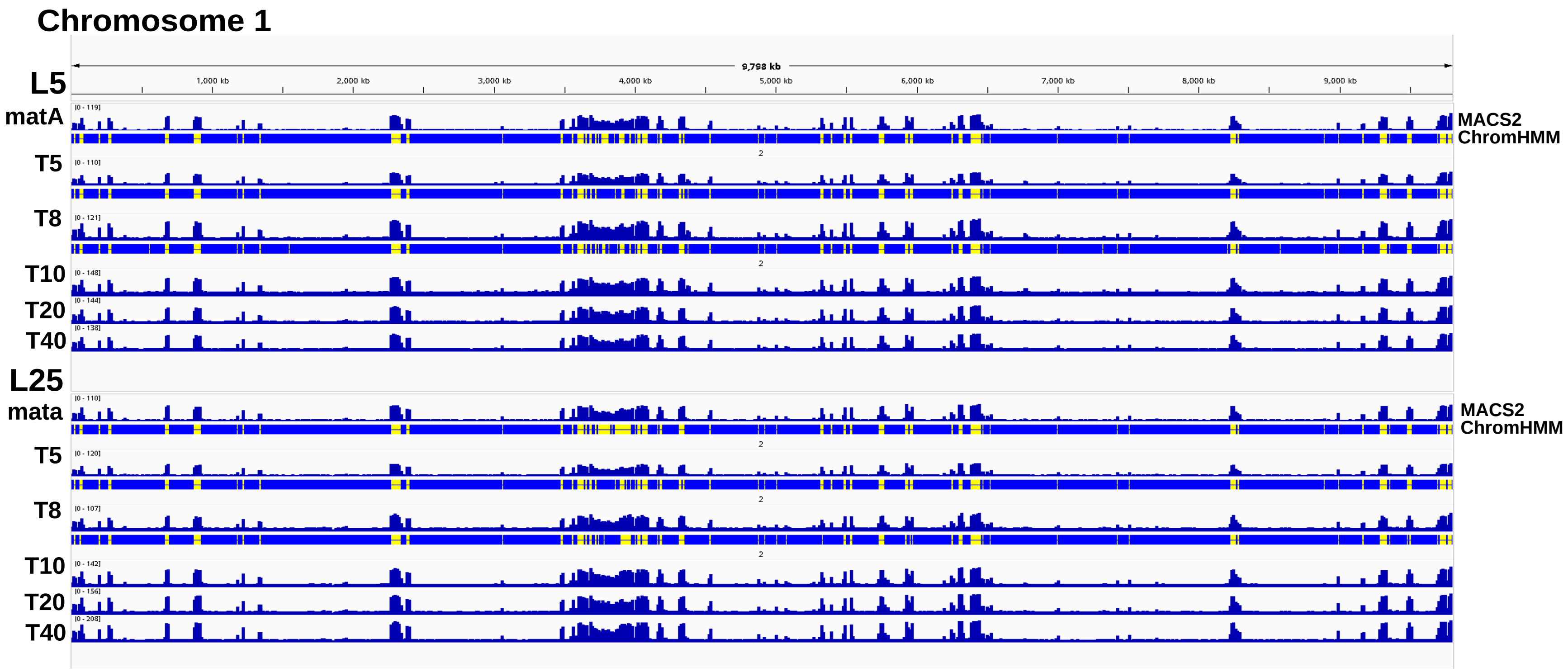

Figure S16: IGV screenshot showing lines 5 and 25 across transfers 5, 8, 10, 20, and 40. In the ancestor and the first two transfers, we include a comparison of two computational methods used to identify H3K9me3-enriched regions. Yellow fragments indicate regions predicted as H3K9me3-enriched by ChromHMM. Chromosome (Linkage Group) I is shown, as a representative example, to illustrate the absence of major changes in H3K9me3 enrichment over time.

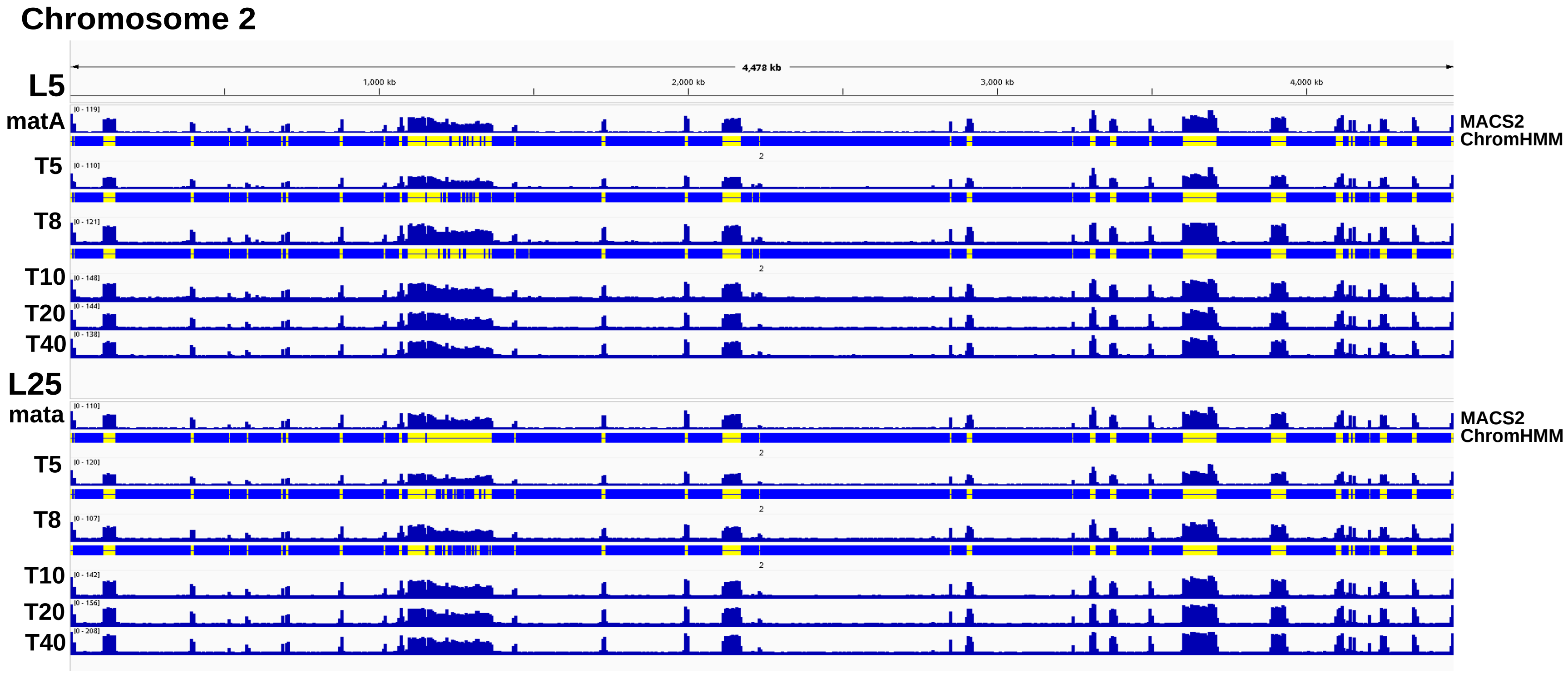

Figure S17: IGV screenshot showing lines 5 and 25 across transfers 5, 8, 10, 20, and 40. In the ancestor and the first two transfers, we include a comparison of two computational methods used to identify H3K9me3-enriched regions. Yellow fragments indicate regions predicted as H3K9me3-enriched by ChromHMM. Chromosome (Linkage Group) II is shown, as a representative example, to illustrate the absence of major changes in H3K9me3 enrichment over time.

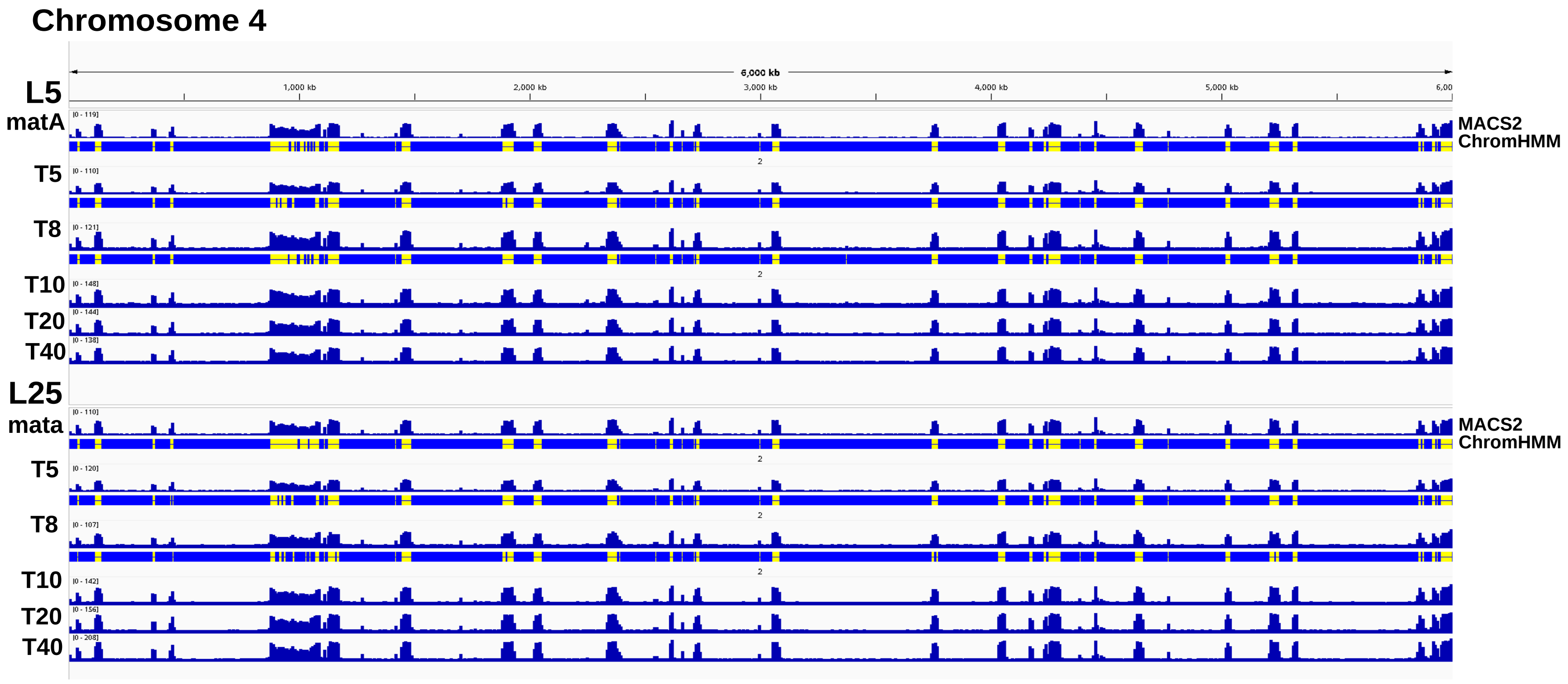

Figure S18: IGV screenshot showing lines 5 and 25 across transfers 5, 8, 10, 20, and 40. In the ancestor and the first two transfers, we include a comparison of two computational methods used to identify H3K9me3-enriched regions. Yellow fragments indicate regions predicted as H3K9me3-enriched by ChromHMM. Chromosome (Linkage Group) IV is shown, as a representative example, to illustrate the absence of major changes in H3K9me3 enrichment over time.

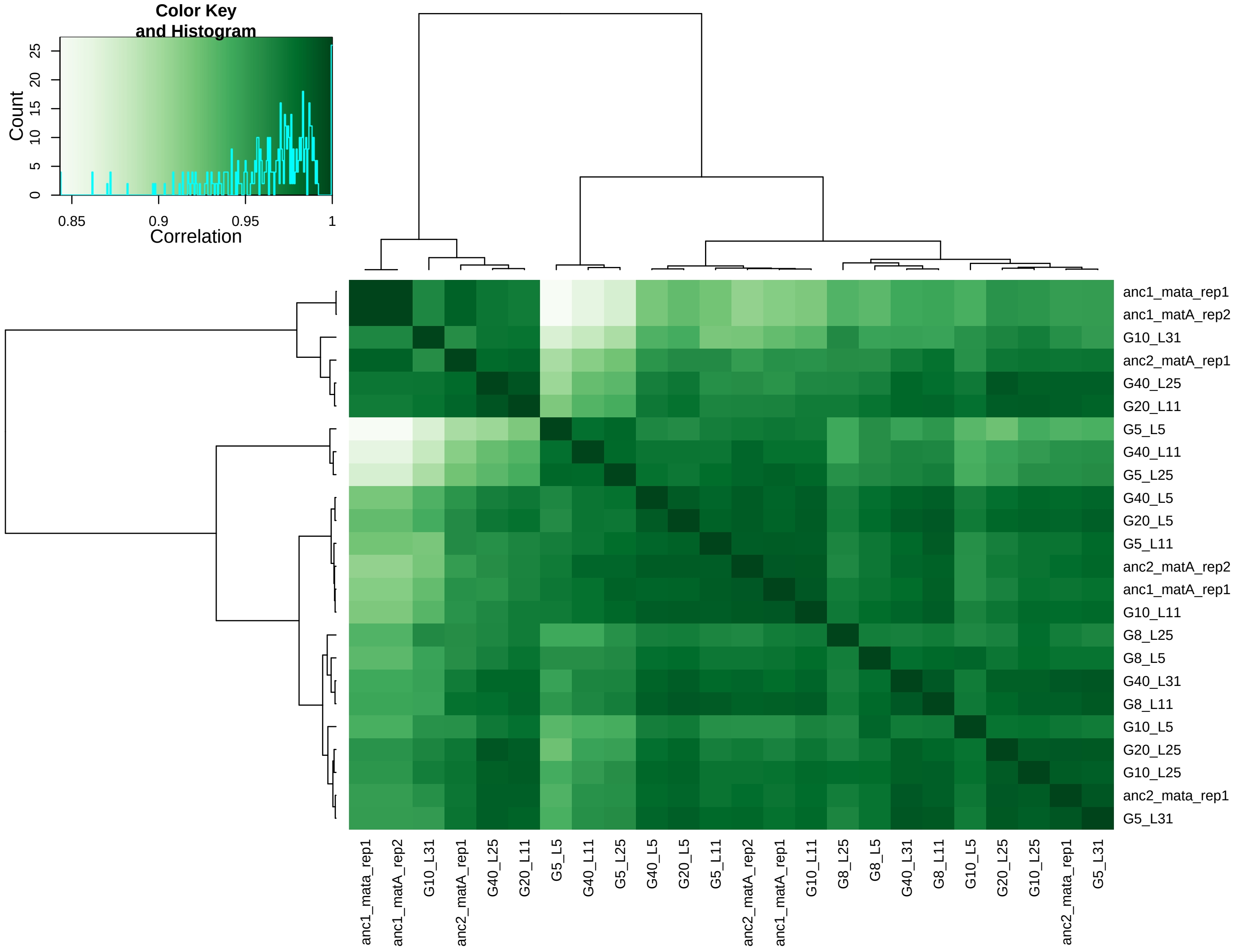

Figure S19: Correlation heatmap of H3K9me3 regions. Created with the package Diffbind. Intensities of color green represent the Pearson coefficient number

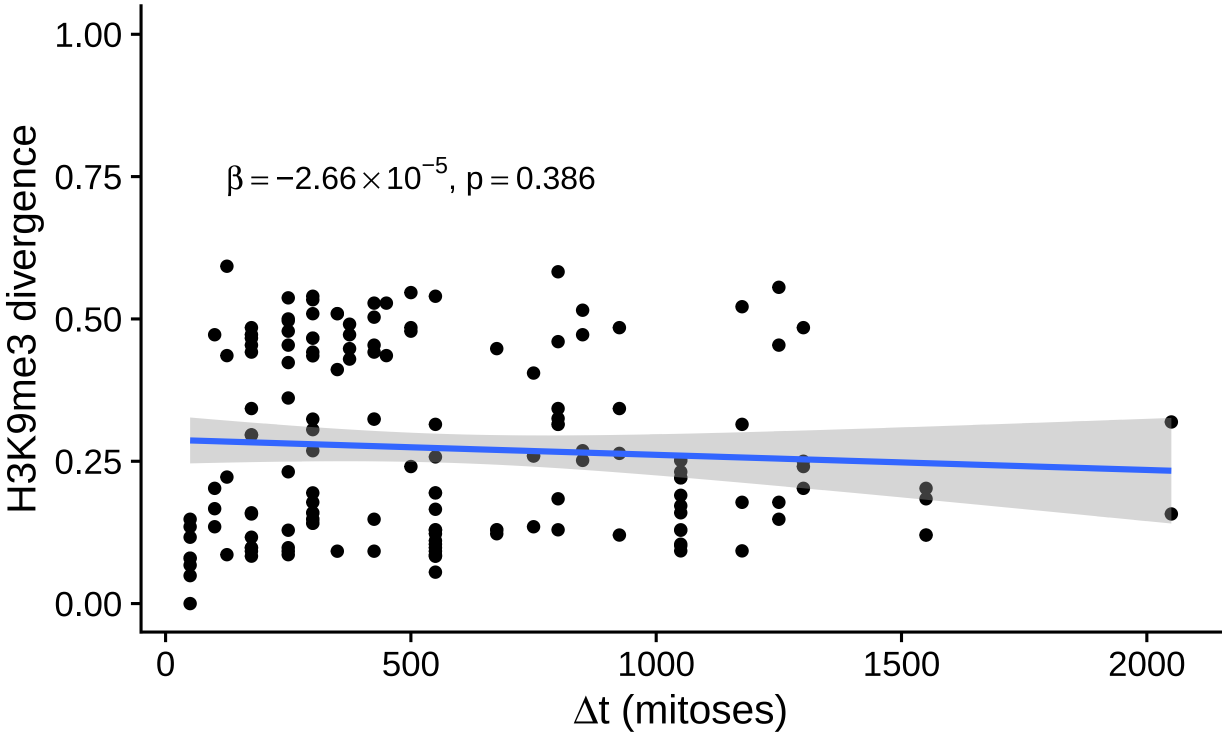

Figure S20: Divergence in presence and absence of peaks of H3K9me3 among the MA lines. The slope of the regression line of divergence in H3K9me3 against number of mitoses separating the MA lines is not statistically different from zero.

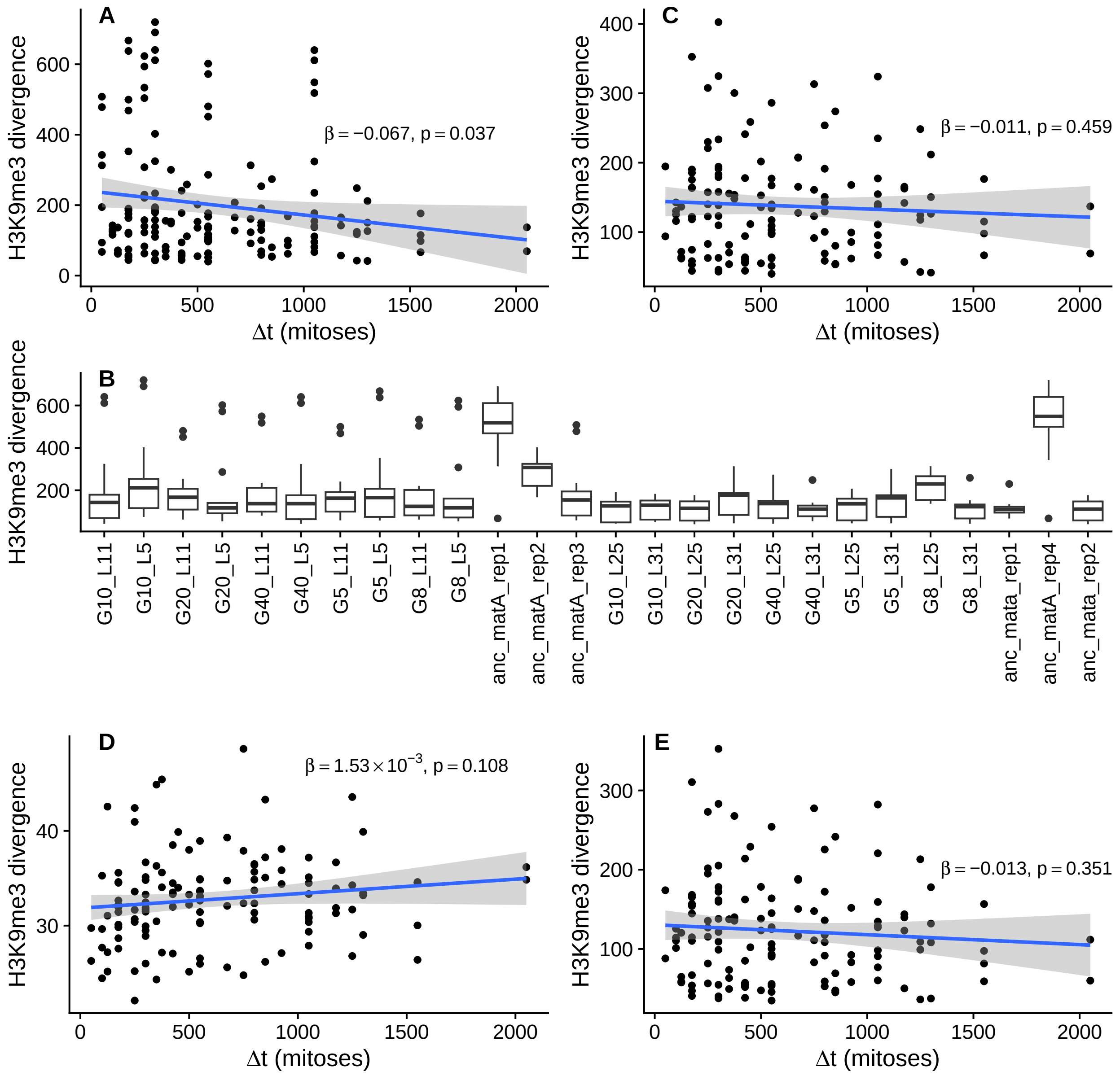

Figure S21: Divergence in H3K9me3 read counts in bins across the genome among the MA lines. A) Data for whole genome in 5kb bins, where a small negative slope was observed. B) Divergence for each sample compared to other samples within pedigree. Two samples, that are replicates of the mat A ancestor, have considerably higher divergence to other samples. C) Reanalysis of divergence across the whole genome, with the two outlier samples detected in B) excluded. Divergence does not change significantly as the MA lines diverge. D) Data for centromeric regions only in 500 bp bins, with two outlier samples are excluded; the slope of divergence is not significantly different from zero. E) Data for regions excluding the centromeres in 5 kb bins, with two outlier samples excluded; the slope of divergence is not significantly different from zero.

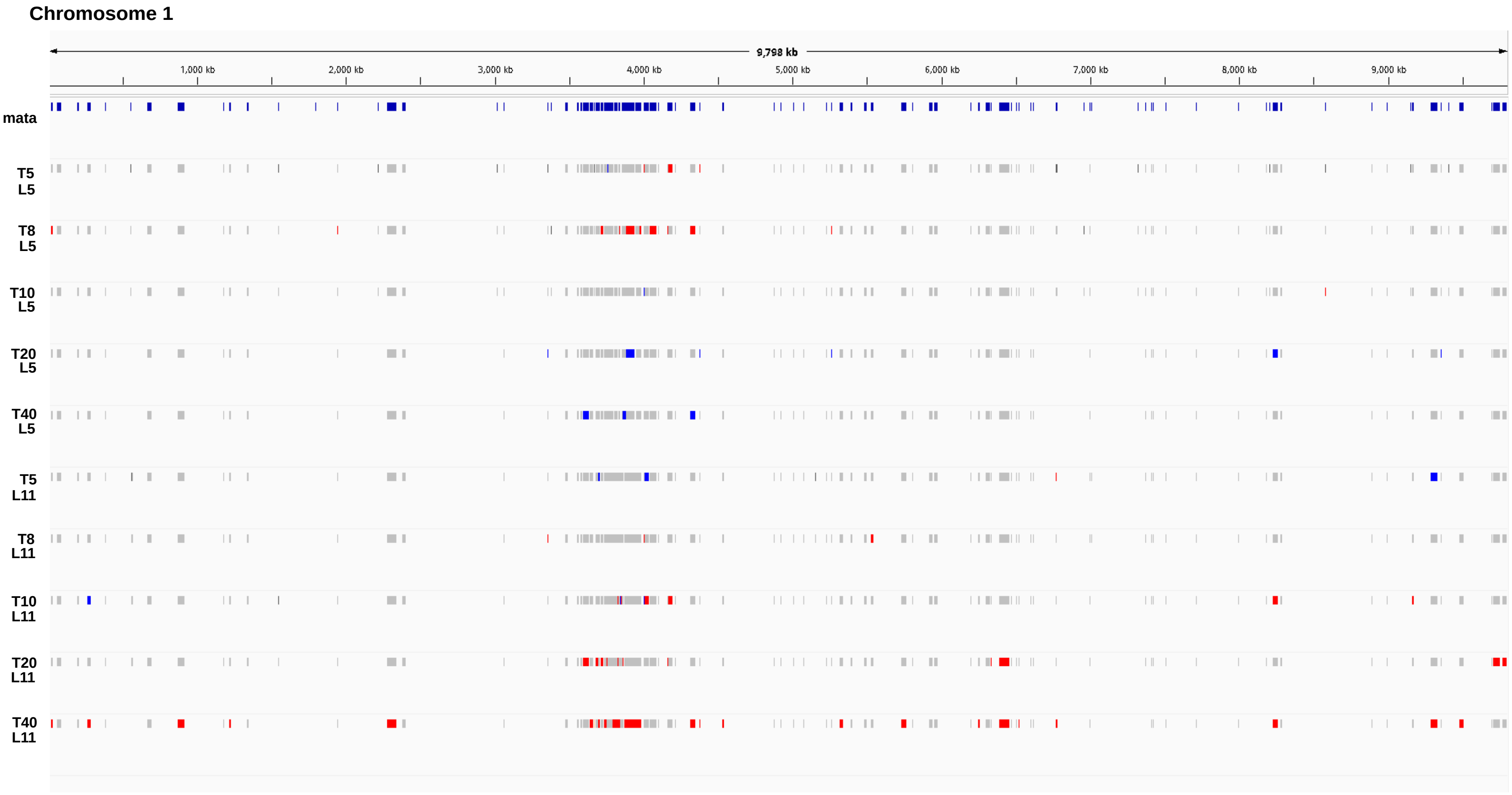

Figure S22: Results of ChromTime analysis. Representative examples showing lines 5 and 11, limited to chromosome 1. Grey regions indicate steady H3K9me3 domains, red denotes regions classified as expanded, and blue indicates regions classified as contracted over time.

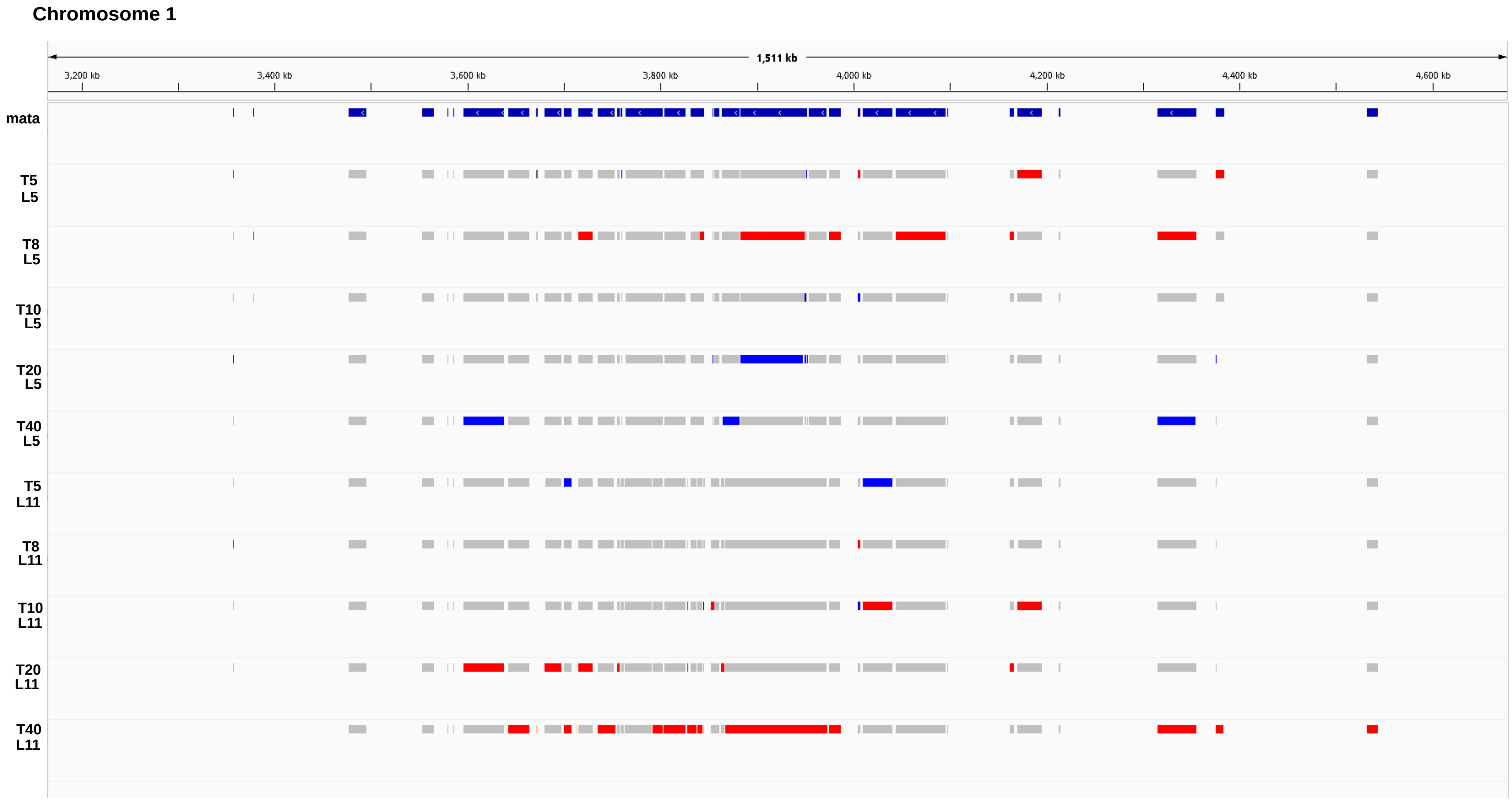

Figure S23: Results of ChromTime analysis. Representative examples showing lines 5 and 11, limited to centromeric regions of chromosome 1. Grey regions indicate steady H3K9me3 domains, red denotes regions classified as expanded, and blue indicates regions classified as contracted over time. Zoom-in on the centromeric region shows that many of the regions labeled as expansions or contractions appear to be technical artifacts, rather than true biological changes.

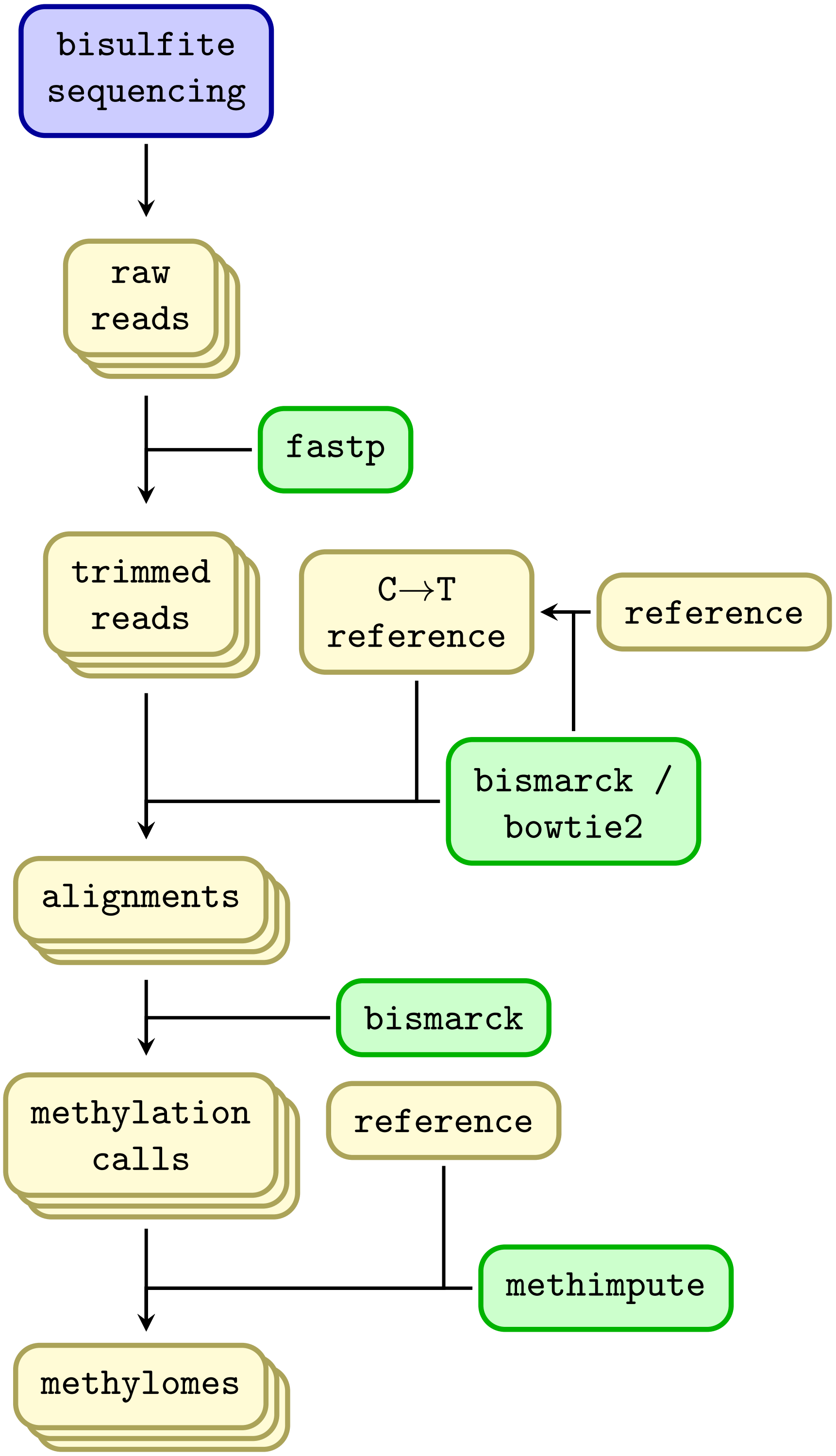

Figure S24: An overview of the bioinformatics workflow for processing WGBS samples. Yellow boxes are files, and green boxes represent programs.

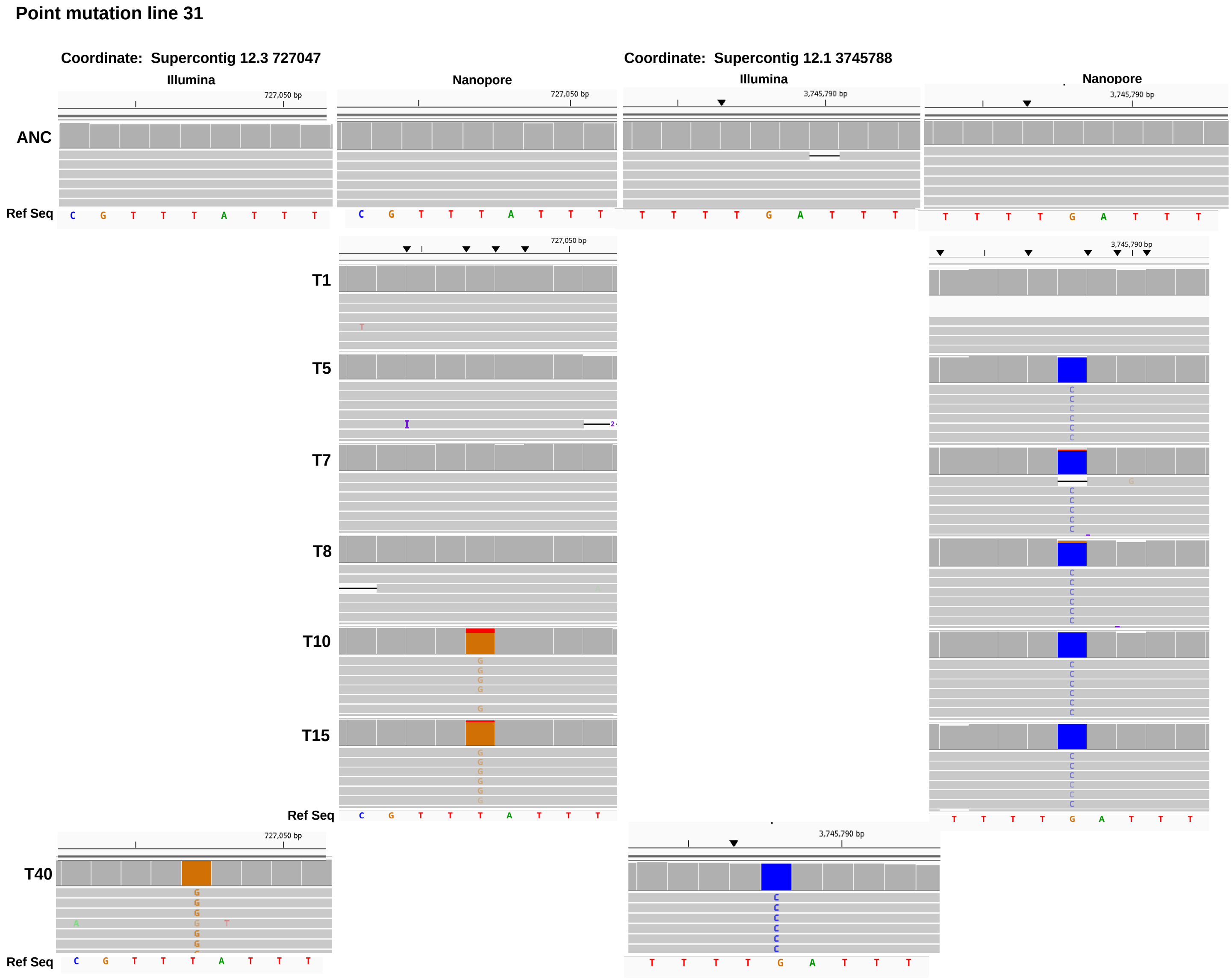

Figure S25: IGV screenshots showing single-nucleotide polymorphisms (SNPs) identified in MA line 31. Shown are representative visualizations of point mutations detected by Nanopore and Illumina sequencing: a T→G substitution in transfer 10 at chromosome 3, position 717,047, and a G→C substitution in transfer 5 at chromosome 1, position 3,745,788. For each mutation, we present screenshots of the ancestor sequenced by Illumina and Nanopore at the mutation coordinate, followed by intermediate transfers sequenced by Nanopore and transfer 40 sequenced by Illumina.

1.4 Supplementary Tables

Table S1: Samples sequenced with bisulfite sequencing. Lines 1 through 20 are descended from the *mat A* ancestor, and lines 21 through 40 from the *mat a* ancestor.

Transfer	Line	Reads	Depth (x)	Coverage (%)	Bs. eff.
Ancestor	*mat A* rep 1	15129280	53		
Ancestor	*mat A* rep 2	14203858	50		
Ancestor	*mat A* rep 3	11905318	41		
Ancestor	*mat a* rep 1	12009736	42		
Ancestor	*mat a* rep 2	13310900	46		
Ancestor	*mat a* rep 3	12958022	45		
5	L1	13352788	47		
5	L6	13224652	46		
5	L7	12854380	45		
5	L10 rep 1	14067628	49		
5	L10 rep 2	14652934	51		
5	L13	14044374	49		
5	L14	14065732	49		
5	L15	13833630	48		
5	L17	13354348	47		
5	L19	11498660	40		
5	L20	14094228	49		
5	L21	13514998	47		
5	L28	13554304	48		
5	L29	12014028	42		
5	L30	12645992	44		
5	L33	14542396	51		
5	L34	15461790	54		
5	L35	13356940	47		
5	L36	9685796	34		
5	L39	14899702	52		
5	L40	15231878	53		
20	L1	16455520	58		
20	L6	16536660	58		
20	L7	16499612	58		
20	L10 rep 1	13837376	48		
20	L10 rep 2	12983524	45		

Continued on next page...

Table S1 – continued from previous page

Transfer	Line	Reads	Depth (x)	Coverage (%)	Bs. eff.
20	L13	13001046	45		
20	L14	13140234	46		
20	L15	1396670	49		
20	L17	18664836	65		
20	L19	11421242	40		
20	L20	14105642	49		
20	L21	17244100	61		
20	L28	16031346	56		
20	L29	19810272	69		
20	L30	16756842	59		
20	L33	16477048	58		
20	L34	13845756	48		
20	L35	18711334	66		
20	L36	18650916	65		
20	L39	12331098	43		
20	L40	12583648	44		
40	L1	14543480	51		
40	L6	15174730	53		
40	L7	12888682	45		
40	L10 rep 1	12976256	45		
40	L10 rep 2	13621346	48		
40	L13	15574080	54		
40	L14	14154158	49		
40	L15	16945084	59		
40	L17	16308294	57		
40	L19	14457268	50		
40	L20	18716914	66		
40	L21	15669808	55		
40	L28	17091676	60		
40	L29	14925692	52		
40	L30	13043906	46		
40	L33	12954644	45		
40	L34	17665520	62		
40	L35	13437214	47		
40	L36	17087492	60		
40	L39	13776582	48		
40	L40	18100634	63		

Table S2: Samples sequenced with Nanopore sequencing. Length is the median Nanopore read length. Lines 2, 5, and 11 are descended from the *mat A* ancestor, and lines 23, 25, and 31 from the *mat a* ancestor.

Transfer	Line	Reads	Length	Coverage (%)	Depth (x)
Ancestor	*mat A*	226810	3554.5	99.97	37.05
Ancestor	*mat a*	307827	3302	99.97	43.69
1	L2	355489	2576	99.97	31.63
1	L5	355210	2844	99.97	34.13
1	L11	470989	2971	99.97	47.59
1	L23	368655	3496	99.97	42.92
1	L25	301674	2782	99.97	27.49
1	L31	318778	3799	99.97	42.48
5	L2	588728	2905	99.97	58.44
5	L11	260810	3133	99.97	26.91
5	L5	532908	3081	99.97	57.96
5	L23	290791	2667	99.97	26.16
5	L25	450272	3306	99.96	51.42
5	L31	266086	3535	99.97	31.67
7	L2	326525	3357	99.97	37.42
7	L5	207747	4098	99.97	34.9
7	L11	233683	3283	99.97	27.29
7	L23	220030	4507	99.97	34.41
7	L25	204732	3552	99.96	25.99
7	L31	391108	3551	99.97	52.36
8	L2	214994	3982	99.97	34.06
8	L5	241171	3514	99.97	33.86
8	L11	215031	4516	99.97	36.36
8	L23	220595	3474	99.96	27.22
8	L25	262599	3653	99.96	38.25
8	L31	232717	3549	99.96	30.15
10	L2	223022	4201	99.97	34.85
10	L5	399996	3563	99.98	53.49
10	L11	197851	4244	99.97	33.29
10	L23	322348	3427	99.96	39.28
10	L25	140407	5228	99.96	28.22
10	L31	371921	3575	99.96	53.51
15	L2	244290	3561	99.97	33.45
15	L5	251497	3724	99.96	37.62
15	L11	203485	3774	99.97	28.9
15	L23	225741	3723	99.97	33.03
15	L25	399885	3511	99.98	53.32
15	L31	167378	3536	99.95	25.6

Table S3: Samples sequenced with ChIP-sequencing with antibody against H3K9me3. Lines 5 and 11 are descened from the *mat A* ancestor, and lines 25 and 31 from the *mat a* ancestor.

Transfer	Line	Reads	Depth (x)
Ancestor	*mat a* rep 1	4406326	13
Ancestor	*mat a* rep 2	8573262	25
Ancestor	*mat A* rep 1	16456278	48
Ancestor	*mat A* rep 2	3734610	11
Ancestor	*mat A* rep 3	4477949	13
Ancestor	*mat A* rep 4	7454673	22
5	L5	3860381	11
5	L11	4643805	13
5	L25	3833040	11
5	L31	7346841	21
8	L5	6753961	20
8	L11	6179668	18
8	L25	6496179	19
8	L31	21930090	64
10	L5	9824968	29
10	L11	5656063	16
10	L25	7835358	23
10	L31	1077318	32
20	L5	6510277	19
20	L11	9720105	29
20	L25	10277927	30
20	L31	23942748	71
40	L5	6019916	18
40	L11	3935274	11
40	L25	11786931	35
40	L31	6697276	20
